# Identification of 7α,24-dihydroxy-3-oxocholest-4-en-26-oic and 7α,25-dihydroxy-3-oxocholest-4-en-26-oic acids in human cerebrospinal fluid and plasma

**DOI:** 10.1016/j.biochi.2018.06.020

**Published:** 2018-10

**Authors:** Jonas Abdel-Khalik, Peter J. Crick, Eylan Yutuc, Andrea E. DeBarber, P. Barton Duell, Robert D. Steiner, Ioanna Laina, Yuqin Wang, William J. Griffiths

**Affiliations:** a Institute of Life Science, Swansea University Medical School, ILS1 Building, Singleton Park, Swansea, SA2 8PP, UK; b Department of Physiology and Pharmacology, Oregon Health and Science University, Portland, OR, USA; c Knight Cardiovascular Institute, Oregon Health and Sciences University, Portland, OR, USA; d Department of Pediatrics, University of Wisconsin School of Medicine and Public Health, Madison, WI, USA; e Athens Medical Group, Athens Medical Center, Marousi Athens, Greece

**Keywords:** Oxysterol, Hereditary spastic paraplegia type 5, Cerebrotendinous xanthomatosis, Cytochrome P450, Bile acid, Brain

## Abstract

Dihydroxyoxocholestenoic acids are intermediates in bile acid biosynthesis. Here, using liquid chromatography – mass spectrometry, we confirm the identification of 7α,24-dihydroxy-3-oxocholest-4-en-26-oic and 7α,25-dihydroxy-3-oxocholest-4-en-26-oic acids in cerebrospinal fluid (CSF) based on comparisons to authentic standards and of 7α,12α-dihydroxy-3-oxocholest-4-en-26-oic and 7α,x-dihydroxy-3-oxocholest-4-en-26-oic (where hydroxylation is likely on C-22 or C-23) based on exact mass measurement and multistage fragmentation. Surprisingly, patients suffering from the inborn error of metabolism cerebrotendinous xanthomatosis, where the enzyme CYP27A1, which normally introduces the (25 R)26-carboxylic acid group to the sterol side-chain, is defective still synthesise 7α,24-dihydroxy-3-oxocholest-4-en-26-oic acid and also both 25 R- and 25 S-epimers of 7α,12α-dihydroxy-3-oxocholest-4-en-26-oic acid. We speculate that the enzymes CYP46A1 and CYP3A4 may have C-26 carboxylase activity to generate these acids. In patients suffering from hereditary spastic paraplegia type 5 the CSF concentrations of the 7α,24- and 7α,25-dihydroxy acids are reduced, suggesting an involvement of CYP7B1 in their biosynthesis in brain.

## Highlights

• Dihydroxy-3-oxocholest-5-en-26-oic are found in human CSF and plasma.
• Hydroxy groups may be at 7α,24, 7α,25, or 7α,12α.
• Another acid is hydroxylated at 7α and in the side-chain probably at C-22 or C-23.
• In patients with CTX acids with 25 R or 25 S stereochemistry are found.
• In patients with SPG5 the concentrations of acids in CSF are reduced.

## Graphical abstract

**Figure:**
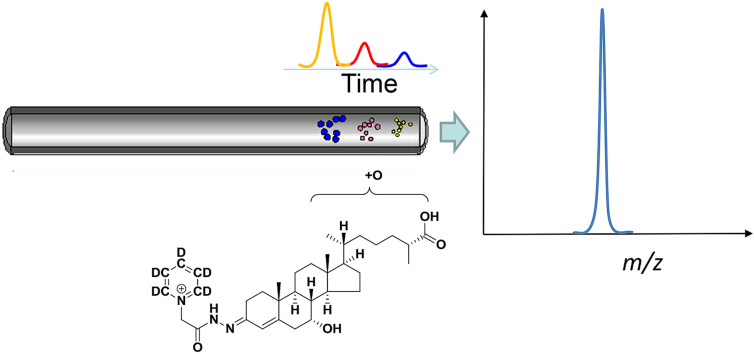

## 1 Introduction

**Fig. 1:**
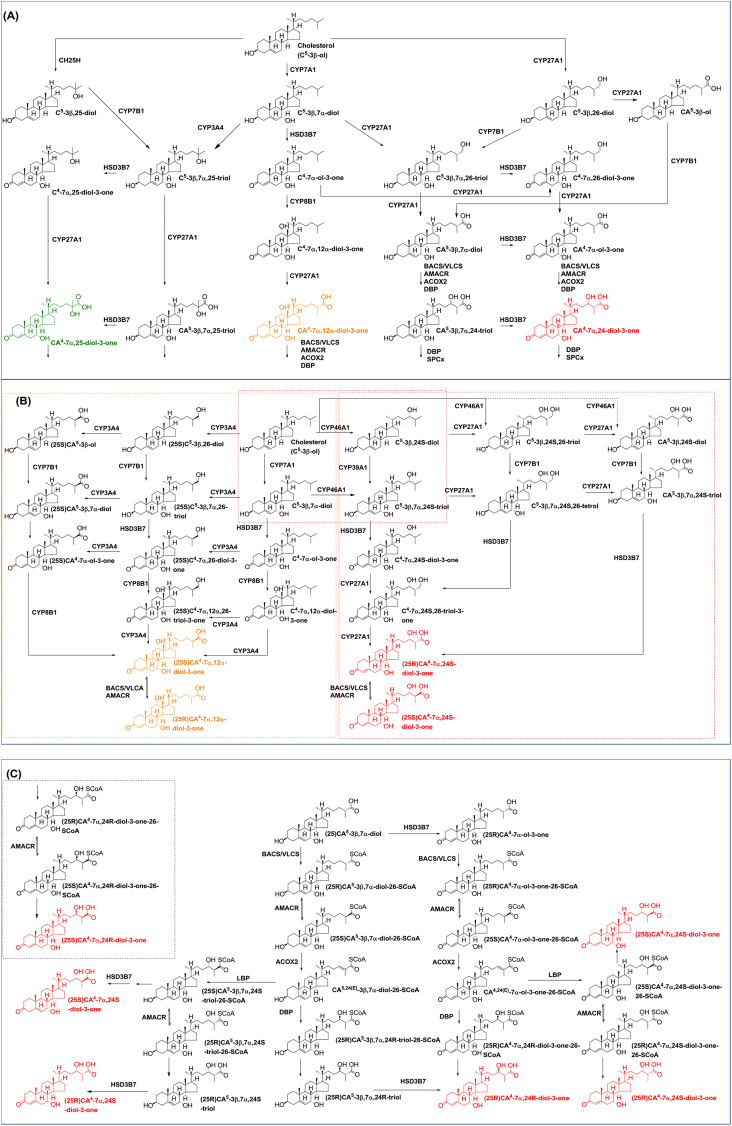
Intermediates in bile acid biosynthesis pathways. (A) Formation of dihydroxy-3-oxocholest-4-en-26-oic acids from cholesterol via the acidic, neutral and 25-hydroxylase pathways [1,2]. In the acidic and neutral pathway stereochemistry at C-25 is assumed to be 25 R unless otherwise indicated. (B) Formation of (25 R)CA^4^-7α,24 S-diol-3-one and (25 S)CA^4^-7α,24 S-diol-3-one via the 24 S-hydroxylase pathway (red box) [21] and of 25 S- and 25 R-epimers of CA^4^-7α,12α-diol-3-one in the absence of CYP27A1 [26] (gold box). For simplicity the formation and hydrolysis of CoA-thioesters in the steps preceding and succeeding C-25 racemisation are not shown. (C) Latter steps of the acidic pathway proceeding mostly in the peroxisome leading to the four diastereomers of CA^4^-7α,24-diol-3-one. In liver the dominant product is (25 R)CA^4^-7α,24 R-diol-3-one. The inset shows the formation of (25 S)CA^4^-7α,24 R-diol-3-one from its CoA-thioester following C-25 racemisation. In CTX the enzyme CYP27A1 is deficient, while in SPG5 CYP7B1 is deficient. Abbreviations: ACOX2: acyl-coenzyme A oxidase 2. AMACR: α-methylacyl-CoA racemase. BACS: bile acid-CoA synthetase. C: Cholestane. CA: Cholestanoic acid. CH25H: Cholesterol 25-hydroxylase. CYP: Cytochrome P450. DBP: D-Bifunctional Protein. HSD3B7: 3β-Hydroxysteroid dehydrogenase type 7. LBP: L-Bifunctional protein. SPCx: Sterol carrier protein x. VLCS: Very long chain Co-A synthetase. The dihydroxy-3-oxocholest-4-en-26-oic acids CA^4^-7α,25-diol-3-one, CA^4^-7α,12α-diol-3-one and CA^4^-7α,24-diol-3-one are colour coded green, gold and red, respectively.

Bile acids are formed from cholesterol via enzyme catalysed reactions through numerous intermediates [1]. These intermediates include cholestenoic acids, unsaturated C_27_ carboxylic acids derived from cholesterol. 3β-Hydroxycholest-5-en-(25 R)26-oic acid (CA^5^-3β-ol), the simplest cholestenoic acid, is be formed by (25 R)26-hydroxylation of cholesterol followed by oxidation to the (25 R)26 carboxylic acid; both reactions catalysed by the enzyme cytochrome P450 (CYP) 27A1 (Fig. 1A). This acid is then hydroxylated by CYP7B1 to give 3β,7α-dihydroxycholest-5-en-(25 R)26-oic acid (CA^5^-3β,7α-diol). Once 7α-hydroxylated, the acid is a substrate for the enzyme hydroxysteroid dehydrogenase (HSD) 3B7, which is a 3β-hydroxysteroid dehydrogenase Δ^5^,Δ^4^ isomerase, giving the product 7α-hydroxy-3-oxocholest-4-en-(25 R)26-oic acid (CA^4^-7α-ol-3-one) [2]. Alternatively, the two 7α-hydroxy acids can be formed from 7α-hydroxycholesterol (C^5^-3β,7α-diol) with (25 R)26-hydroxylation and carboxylation preceding or succeeding oxidation and isomerisation in the ring system (Fig. 1A). In 2010 Ogundare et al. reported the detection of CA^5^-3β-ol and CA^4^-7α-ol-3-one in human cerebrospinal fluid (CSF) and also partially identified two dihydroxy-3-oxocholest-4-en-26-oic acids [3]. At that time authentic standards were unavailable to exactly identify the latter two acids. Ogundare et al. were able to identify C-7 as one of the sites of hydroxylation but made only tentative suggestions of the locations of the second hydroxy groups [3]. Since the study by Ogundare et al. [3] others have confirmed the presence of CA^5^-3β-ol and CA^4^-7α-ol-3-one in human CSF [4,5]. It is noteworthy that CA^4^-7α-ol-3-one was detected by Nagata et al. in chronic subdural hematoma, but analytical methods of the time were insufficient to detect the acid in normal CSF [6], although Axelson et al. had found it to be a normal constituent of human blood [7]. Note, with the exception of 25-hydroxy acids, unless specifically stated otherwise stereochemistry at C-25 is assumed to be 25 R.

Cholestenoic acids with a 3β-hydroxy-5-ene structure have been shown to be ligands to the liver X receptors (LXRs) α and β, CA^5^-3β,7α-diol displaying neuroprotective properties while its epimer CA^5^-3β,7β-diol and CA^5^-3β-ol being neurotoxic [3,8,9]. CA^4^-7α-ol-3-one is not a ligand to LXR, and the biological properties of down-stream acids are unknown [9].

Here we describe the identification and quantification, using enzyme-assisted derivatisation and liquid chromatography (LC) - high resolution mass spectrometry (HRMS) with multistage fragmentation (MS^n^), of the dihydroxy-3-oxocholest-4-en-26-oic acids reported earlier by Ogundare et al. in human CSF [3]. The current study goes beyond that of Ogundare et al. [3], Saeed et al. and Schöls et al. [4,5] in that we have specifically targeted multiply hydroxylated acids, exploiting synthetic standards to confirm, where possible, identifications made by LC-HRMS(MS^n^) and performed quantification in CSF and in plasma.

## 2 Materials and methods

### 2.1 Materials

**Fig. 2:**
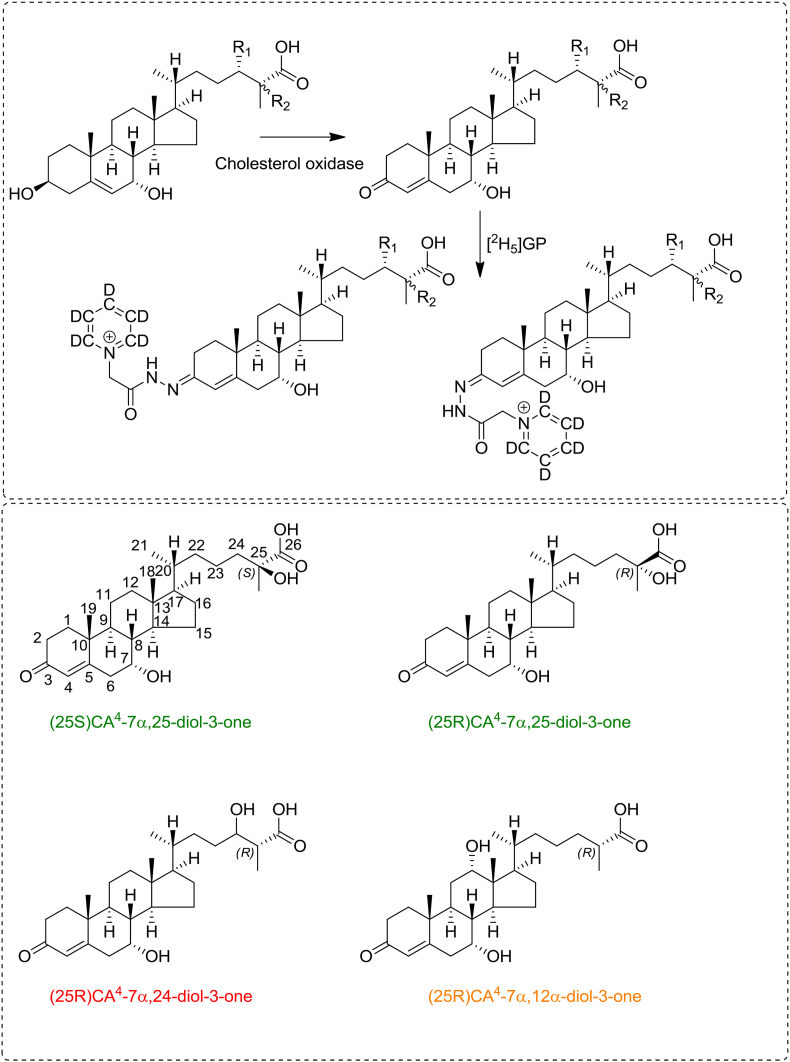
Conversion of 3β-hydroxy-5-ene acids to their 3-oxo-4-ene analogues by cholesterol oxidase enzyme from *Streptomyces* Sp and derivatisation with [^2^H_5_]GP to give *syn* and *anti* conformers. In CA^5^-3β,7α,24 S-triol and CA^4^-7α,24 S-diol-3-one R_1_ = OH and R_2_ = H. In CA^5^-3β,7α,25-triol and CA^4^-7α,25-diol-3-one R_1_ = H and R_2_ = OH (upper panel). Structures of (25 S)CA^4^-7α,25-diol-3-one and its isomers (25 R)CA^4^-7α,25-diol-3-one, (25 R)CA^4^-7α,24-diol-3-one and (25 R)CA^4^-7α,12α-diol-3-one are shown in the lower panel.

The reagents and solvents were as described in Abdel-Khalik et al. [10]. The authentic standards of 3β,7α,24 S-trihydroxycholest-5-en-(25 R)26-oic acid ((25 R)CA^5^-3β,7α,24 S-triol) and 3β,7α,25-trihydroxycholest-5-en-(25 R/S)26-oic acid (CA^5^-3β,7α,25-triol) were from Avanti Polar Lipids Inc (Alabaster, AL). Their 3-oxo-4-ene analogues, 7α,24 S-dihydroxy-3-oxocholest-4-en-(25 R)26-oic acid ((25 R)CA^4^-7α,24 S-diol-3-one) and 7α,25-dihydroxy-3-oxocholest-4-en-(25 R/S)26-oic acid (CA^4^-7α,25-diol-3-one), were prepared by treatment with cholesterol oxidase from *Streptomyces* sp (Sigma-Aldrich, Gillingham, Dorset UK) (Fig. 2).

All CSF and plasma samples were provided with written informed consent and institutional review board (IRB) approval and were collected according to the principles of the Declaration of Helsinki. Control CSF (n = 42) was from previous studies performed in Swansea, collected from subjects with no known genetic defect in cholesterol biosynthetic or metabolic enzymes. CSF from cerebrotendinous xanthomatosis (CTX, n = 6) patients was collected as part of an IRB-approved study at Oregon Health & Science University (OHSU), Portland, OR (see Supplemental Table 1 for mutation data). Plasma from CTX (n = 13) and CSF (n = 3) and plasma (n = 2) from hereditary spastic paraplegia type 5 (SPG5) patients were from earlier studies in Swansea [9] or provided by the Athens Medical Center. Control plasma (n = 24), from subjects with no known genetic defect in cholesterol biosynthetic or metabolic enzymes, was from a previous study in Swansea [10].

### 2.2 Sample preparation and analysis

Two hundred and fifty μL of CSF was added dropwise to 2.1 mL of ethanol containing 2 ng of [^2^H_7_]24(R/S)-hydroxycholesterol, 2 ng [^2^H_7_]22 R-hydroxycholest-4-en-3-one, 2 ng [^2^H_7_]7α-hydroxycholesterol, 1.38 ng [^2^H_6_]7α,25-dihydroxycholesterol, 4 ng [^2^H_6_]25-hydroxyvitamin D_3_ and 800 ng [^2^H_7_]cholesterol. Each sample was sonicated for 5 min, after which 0.65 mL of water was added dropwise while continuing to sonicate. Sonication was continued for a further 5 min. The solution was centrifugation at 2400 g, 4 °C, for 30 min. The supernatant, now in 3 mL of 70% ethanol, was loaded onto a 200 mg Certified Sep-Pak C_18_ column (Waters, Elstree, Herts, UK). The 3 mL eluate was combined with a column wash of 4 mL of 70% ethanol to give SPE1-Fr1 (7 mL) in which oxysterols including cholestenoic acids elute.

The oxysterol fraction was then derivatised and analysed as described in Abdel-Khalik et al. [10]. In brief, the oxysterol fraction above was divided into two equal sub-fractions A and B and lyophilised. Each sub-fraction was reconstituted in 100 μL of propan-2-ol. One mL of 50 mM phosphate buffer pH 7 containing 0.26 u of cholesterol oxidase from *Streptomyces* sp (Sigma-Aldrich, Gillingham, Dorset, UK) was added to sub-fraction A and incubated for 1 h at 37 °C. The reaction was quenched with 2 mL of methanol. Sub-fraction B was treated in an identical fashion but in the absence of cholesterol oxidase. To each sub-fraction 150 μL of glacial acetic acid was added followed by 190 mg of [^2^H_5_]Girard P (GP) reagent as the bromide salt to sub-fraction A and 150 mg of [^2^H_0_]GP as the chloride salt to sub-fraction B. The reaction proceeded at room temperature overnight in the dark. Excess derivatisation reagent was then removed by re-cycling solid phase extraction (SPE) on a 60 mg Oasis HLB column (Waters, Elstree, Herts, UK). Each eluate was diluted to half its organic content until 17.5% organic was achieved at which point derivatised oxysterols including cholestenoic acids were bound to the column and unreacted GP reagent was in the flow-through. After a wash with 10% methanol, derivatised oxysterols were eluted in 2 mL of methanol. LC-HRMS(MS)^n^ was performed as described in Abdel-Khalik et al. [10] using a Dionex Ultimate 3000 L C system (Dionex now Thermo Fisher Scientific, Hemel Hempstead, UK) with a C_18_ Hypersil Gold column (Thermo Fisher Scientific) and Orbitrap Elite mass spectrometer (Thermo Fisher Scientific). Quantification was by the isotope dilution method exploiting LC-HRMS data with [^2^H_7_]24(R/S)-hydroxycholesterol and [^2^H_7_]22 R-hydroxycholest-4-en-3-one as internal standards for sub-fractions A (with cholesterol oxidase) and B (without cholesterol oxidase), respectively. Plasma samples were prepared and analysed as described in Abdel-Khalik et al. [10] in a similar manner to described above for CSF. Quality control (QC) samples were prepared for plasma and CSF by combining aliquots of control samples.

## 3 Results

### 3.1 Dihydroxy-3-oxocholest-4-en-26-oic acids in CSF

#### 3.1.1 CA^4^-7α,24-diol-3-one

**Fig. 3:**
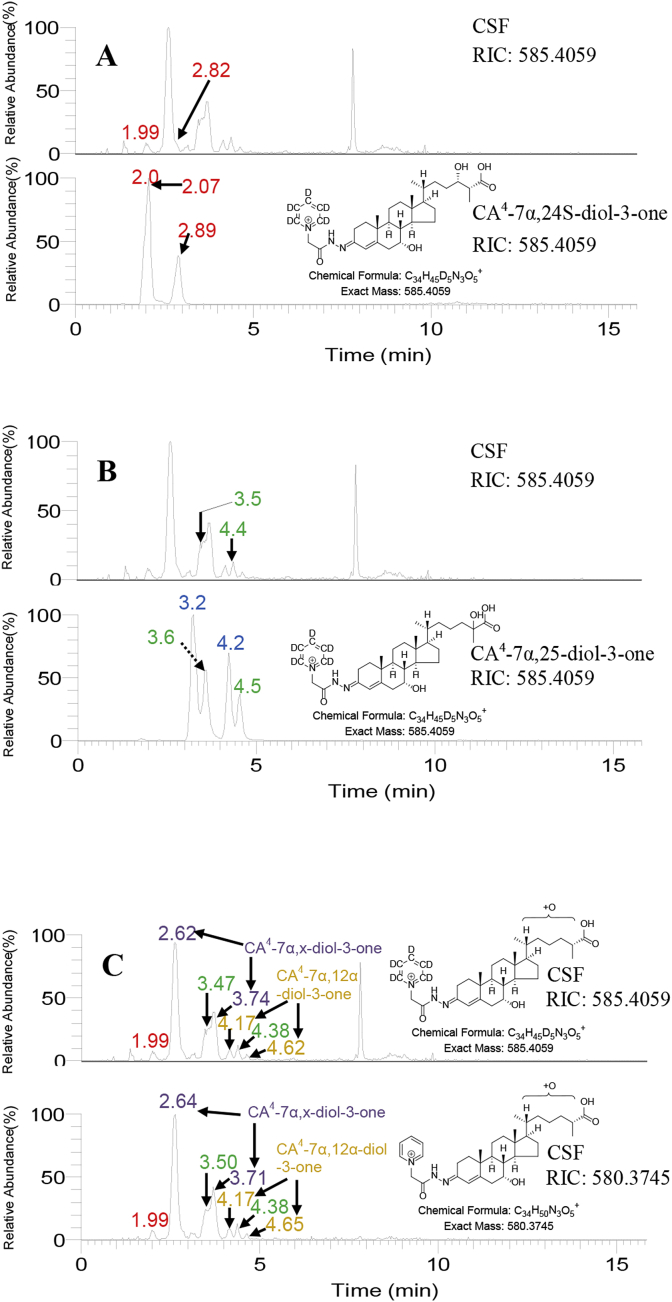
LC-HRMS RICs (585.4059 ± 10 ppm) corresponding to [M]^+^ ions of [^2^H_5_]GP derivatised dihydroxy-3-oxocholest-4-en-26-oic acids. (A) Control CSF (upper panel), authentic standard of (25 R)CA^4^-7α,24 S-diol-3-one (lower panel). (B) Control CSF (upper panel), authentic standard of (25 R/S)CA^4^-7α,25-diol-3-one (lower panel). (C) Control CSF (SPE1-Fr1A) treated with cholesterol oxidase followed by [^2^H_5_]GP reagent (upper panel), control CSF (SPE1-Fr1B) treated with [^2^H_0_]GP reagent in the absence of cholesterol oxidase (lower panel). In (C) the RICs for [^2^H_5_]GP derivatised and [^2^H_0_]GP derivatised acids are plotted with the same y-axis. Data was acquired in the Orbitrap analyser of the Orbitrap Elite instrument. Peaks corresponding to the dihydroxy-3-oxocholest-4-en-26-oic acids CA^4^-7α,25-diol-3-one, CA^4^-7α,12α-diol-3-one, CA^4^-7α,24-diol-3-one and CA^4^-7α,x-diol-3-one are colour coded green, gold, red and purple respectively.

The authentic standard of [^2^H_5_]GP-derivatised (25 R)CA^4^-7α,24 S-diol-3-one gave rise to two peaks in the LC-HRMS reconstructed ion chromatogram (RIC) for its [M]^+^ ion at *m/z* 585.4059, corresponding to the *syn* and *anti* conformers of the derivative (Fig. 3A, lower panel). The more abundant conformer eluted at almost the same retention time as a chromatographic peak in the equivalent LC-HRMS RIC from human CSF (1.99 min, Fig. 3A, upper panel). The less abundant conformer was observed as a shoulder (2.82 min) to an unknown peak, CA^4^-7α,x-diol-3-one, in the CSF sample (Fig. 3A). Note during the course of this study there was some retention time drift as the recording of data was spread over many months. However, there was no change in the order of elution of cholestenoic acids as assessed by recording of QC samples within each batch of samples.

**Fig. 4:**
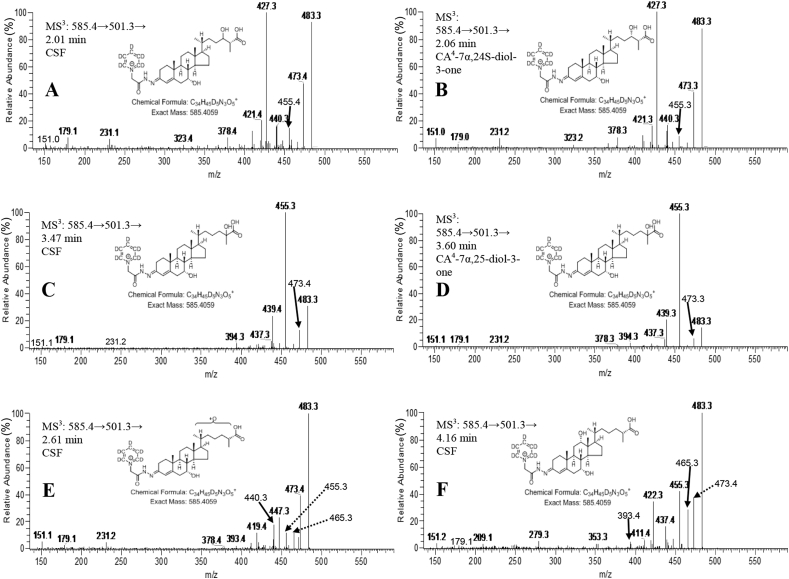
MS^3^ ([M]^+^→[M-Py]^+^→, i.e. 585.4→501.3→) spectra acquired in the analyses of control CSF (A, C, E & F) and authentic standards of (25 R)CA^4^-7α,24 S-diol-3-one (B) and (25 R/S)CA^4^-7α,25-diol-3-one (D). The spectra confirm the identification of CA^4^-7α,24-diol-3-one in (A), of CA^4^-7α,25-diol-3-one in (C), of CA^4^-7α,x-diol-3-one in (E) and of CA^4^-7α,12α-diol-3-one in (F). Data was acquired in the linear ion trap (LIT) analyser of the Orbitrap Elite hybrid instrument with an accuracy of *m*/*z* ± 0.1 for most fragment-ions.

**Fig. 5:**
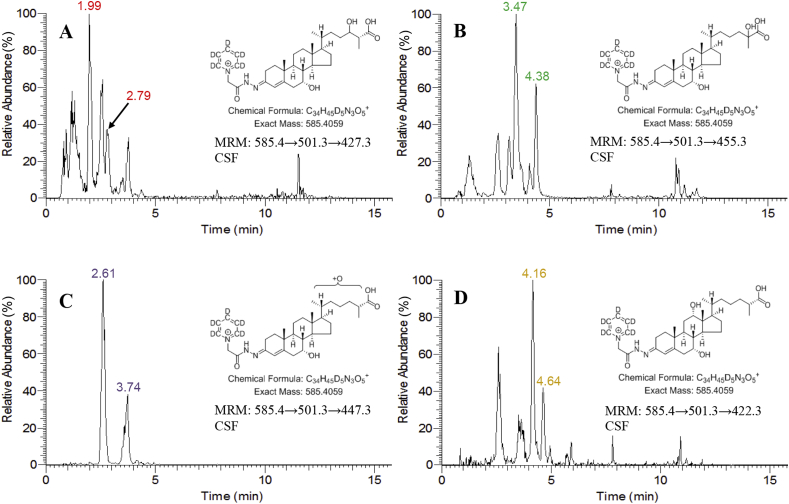
LC-MS^3^ MRM transitions appropriate to (A) CA^4^-7α,24-diol-3-one, (B) CA^4^-7α,25-diol-3-one, (C) CA^4^-7α,x-diol-3-one and (D) CA^4^-7α,12α-diol-3-one from the analysis of a control CSF sample. MRMs were generated from data acquired in the LIT analyser of the Orbitrap Elite hybrid instrument with an *m/z* window of ±0.4. Peaks are colour coded as in Fig. 3.

When fragmented GP-derivatised sterols give intense [M-Py]^+^ ions due to loss of the pyridine (Py) ring (see Supplemental Fig. 1) [11]. The [M-Py]^+^ ion when fragmented further gives structure specific MS^3^ spectra. The MS^3^ ([M]^+^→[M-Py]^+^→, i.e. 585.4→501.3→) spectra acquired at 2.01 min from the CSF sample (Fig. 4A) and 2.06 min from the authentic standard of (25 R)CA^4^-7α,24 S-diol-3-one (Fig. 4B) were essentially identical. A base peak fragment-ion at *m/z* 427.3 is characteristic for the GP-derivatised CA^4^-7α,24 S-diol-3-one structure. Note, MS^3^ spectra were generated in the ion-trap and fragment-ion measurements were accurate to *m/z* ± 0.1 in most cases, hence, fragment-ion *m/z* data is given to only one decimal place. By generating a multiple reaction monitoring (MRM)-like chromatogram 585.4→501.3→427.3 the *syn* and *anti* conformers of CA^4^-7α,24-diol-3-one are resolved from the chemical background of the CSF sample, the conformers are evident as peak at 1.99 and 2.79 min in Fig. 5A. The fragment-ion *m/z* 427.3, probably results from loss of CO_2_ + H_2_ (46 Da) from the [M-Py-CO]^+^ ion at *m/z* 473.3 via a classical charge-remote fragmentation mechanism [12] (Supplemental Fig. 1).

By analysing oxysterol sub-fractions A and B where sample is treated with or without cholesterol oxidase (Fig. 3C upper and lower panels, respectively) 3β-hydroxy-5-ene sterols can be deconvoluted from their 3-oxo-4-ene analogues. In the CSF samples analysed about 90% of the acids were in the CA^4^-7α,24-diol-3-one form, about 10% as CA^5^-3β,7α,24-triol. In the present study the concentration of CA^4^-7α,24-diol-3-one was determined to be 0.3 ± 0.1 ng/mL (mean ± standard deviation, SD, n = 42, see Table 1). In the absence of an isotope labelled authentic standard for the CA^4^-7α,24-diol-3-one, quantification was against the internal standard [^2^H_7_]22 R-hydroxycholest-4-en-3-one. The limit of quantification was 0.05 ng/mL. In a separate study using identical methodology the concentration of CA^4^-7α,24-diol-3-one was found to be 0.4 ± 0.1 ng/mL, n = 15 [10]. While the current methodology can differentiate between 7α- and 7β-hydroxy epimers, 24 S- and 24 R-hydroxy epimers and (25 R)26- and (25 S)26-carboxylate epimers [13], the availability of an authentic standards of just one diastereomer does not allow the definitive stereochemical identification of CA^4^-7α,24-diol-3-one or CA^5^-3β,7α,24-triol.

**Table 1:** Concentrations of dihydroxy-3-oxocholest-4-en-26-oic acids in CSF.

Compound	Control CSF, n = 42			CTX CSF, n = 6				SPG5 CSF, n = 3			
	Mean	Median	SD	Mean	Median	SD	Significance^1^	Mean	Median	SD	Significance^1^
	ng/mL	ng/mL	ng/mL	ng/mL	ng/mL	ng/mL	CTX v Control	ng/mL	ng/mL	ng/mL	SPG5 v Control
CA^4^-7α,24-diol-3-one	0.30	0.30	0.08	0.10	0.10	0.02	P < 0.01	0.07	0.09	0.07	P < 0.01
CA^4^-7α,x-diol-3-one	5.83	5.65	1.49	ND	ND	NA	P < 0.01	0.60	0.27	0.66	P < 0.01
CA^4^-7α,25-diol-3-one	1.60	1.51	0.44	ND	ND	NA	P < 0.01	0.19^2^	0.05	0.27	P < 0.01
(25 R)CA^4^-7α,12α-diol-3-one	1.11	0.82	1.10	0.49	0.29	0.60	P < 0.05	0.64	0.72	0.17	P > 0.05
(25 S)CA^4^-7α,12α-diol-3-one	ND	ND	NA	0.12	0.09	0.11	P < 0.01	ND	ND	NA	NA

Quantification was made utilising LC-HRMS RICs with [^2^H_7_]22 R-hydroxycholest-4-en-3-one as the internal standard.

1. Mann-Whitney test.

2. From Ref. [9].

ND = not detected.

NA = not available.

#### 3.1.2 CA^4^-7α,25-diol-3-one

The authentic standard of CA^5^-3β,7α,25-triol was available only as a racemic mixture of 25 R- and 25 S-epimers. When treated with cholesterol oxidase to give CA^4^-7α,25-diol-3-one and derivatised with [^2^H_5_]GP, four chromatographic peaks were observed in the LC-HRMS RIC for the [M]^+^ ion at *m/z* 585.4059 (Fig. 3B, lower panel). These peaks correspond to the *syn* and *anti* conformers of the 25 R- and 25 S-epimers (Fig. 2). When CSF was analysed there was clear evidence for the presence of CA^4^-7α,25-diol-3-one by the appearance of a shoulder at 3.47 min and a distinct peak at 4.38 min (Fig. 3B, upper panel). To enhance sensitivity, when CSF was prepared for LC-HRMS the final SPE eluate was lyophilised and reconstituted in 90 μL of 60% methanol prior to injection on the LC system. This resulted in a small retention time shift in comparison to the authentic standard which was prepared in the absence of matrix.

The MS^3^ ([M]^+^→[M-Py]^+^→, i.e. 585.4→501.3→) spectra acquired at 3.47 min for CSF (Fig. 4C) and at 3.60 min for the authentic standard of CA^4^-7α,25-diol-3-one (Fig. 4D) are essentially identical. A base peak fragment-ion at *m/z* 455.3 is characteristic of GP-derivatised CA^4^-7α,25-diol-3-one. By recording an MRM chromatogram 585.4→501.3→455.3 for control CSF (Fig. 5B), two distinct peaks are observed at 3.47 and 4.38 min allowing the resolution of CA^4^-7α,25-diol-3-one from other isomers. The fragment-ion at *m/z* 455.3 is, however, not unique to CA^4^-7α,25-diol-3-one (Fig. 4), but the MS^3^ spectra recorded for peaks at 3.47 (Figs. 4C) and 4.38 min confirmed their origin to be CA^4^-7α,25-diol-3-one. The fragment-ion at *m/z* 455.3 results from the loss of H_2_CO_2_ from the [M-Py]^+^ ion, this is likely to be through (i) a combination of dehydration (−18 Da) and decarbonylation (−28 Da) with loss of CO from the GP reagent and loss of the labile 25-hydroxy or 7α-hydroxy group, and/or (ii) loss of the C_26_ acid group as H_2_O plus CO with the second hydrogen abstracted from the C-25-hydroxy group. This reaction could proceed via a 6-electron classical charge-remote fragmentation mechanism [12] (Supplemental Fig. 2). As was the situation with 7α,24-dihydroxy acids, the majority (>90%) of the 7α,25-dihydroxy acid was found to be as the 3-oxo-4-ene i.e. CA^4^-7α,25-diol-3-one. In the current study the concentration of CA^4^-7α,25-diol-3-one in CSF was determined to be 1.6 ± 0.4 ng/mL (Table 1). Quantification was against the internal standard [^2^H_7_]22 R-hydroxycholest-4-en-3-one. The limit of quantitation was 0.02 ng/mL. In an independent study using the same method the level of CA^4^-7α,25-diol-3-one in CSF was found to be 3.0 ± 1.1 ng/mL [10].

#### 3.1.3 CA^4^-7α,x-diol-3-one

Two peaks, probably corresponding to *syn* and *anti* conformers, of a more abundant but unknown dihydroxyoxocholestenoic acid isomer, CA^4^-7α,x-diol-3-one, were detected at 2.62 and 3.74 min in the LC-HRMS RIC from the CSF sample shown in Fig. 3C. As with the 7α,24- and 7α,25-dihydroxy acids, the unknown compound was predominantly in the 3-oxo-4-ene form. Its concentration was determined here to be 5.8 ± 1.5 ng/mL (Table 1) and in an earlier study 9.2 ± 3.0 ng/mL [10]. Quantification was against the internal standard [^2^H_7_]22 R-hydroxycholest-4-en-3-one, assuming a similar response factor to the authentic standards of the 7α,24 S- and 7α,25-dihydroxy acids. Based on this assumption the limit of quantification was 0.05 ng/mL. The MS^3^ ([M]^+^→[M-Py]^+^→) spectrum acquired at 2.61 min (Fig. 4E) shows some similarity to the fragmentation pattern to CA^4^-7α,24-diol-3-one (Fig. 4A and B) and CA^4^-7α,25-diol-3-one (Fig. 4C and D). The pattern of fragment ions at *m/z* 151.1 (*b_1_-12), 179.1 (*b_3_-28) and 231.1 (*c_2_-18 + 2) is characteristic of GP-derivatised 7-hydroxy-3-oxo-4-ene sterols, while in the high *m/z* range the neutral losses [M-Py-18]^+^ (*m/z* 483.3), [M-Py-28]^+^ (*m/*z 473.3), [M-Py-46]^+^ (*m/z* 455.3) are common to GP-derivatised acids (Supplemental Fig. 3). A triad of fragment-ions at *m/z* 419.3 ([M-Py-82]^+^), 440.3 (M-Py-61) and 447.3 ([M-Py-54) are distinctive of the unknown compound, CA^4^-7α,x-diol-3-one, (Fig. 4E). These fragment-ions are suggested to correspond to loss of H_2_CO_2_ and 2 × H_2_O (−82 Da); CO, NH, and H_2_O (−61 Da), and 3 × H_2_O (−54 Da) from the [M-Py]^+^ ion, respectively. A further fragment-ion was observed at 465.3 [M-Py-36]^+^ indicating the presence of at least two labile hydroxy groups, supporting the suggestion of a dihydroxy-3-oxocholest-4-en-26-oic acid structure. While one of the hydroxy groups is at C-7, most likely of 7α-stereochemistry, the location of the second hydroxy group is more difficult to assign possibly at C-22, C-23 or less likely at C-27 (in Supplemental Fig. 3 we have assigned the second hydroxy group to C-23).

#### 3.1.4 CA^4^-7α,12α-diol-3-one

A second pair of peaks corresponding to an unknown dihydroxyoxocholestenoic acid were detected at 4.17 min and 4.62 min in the LC-HRMS RIC shown in Fig. 3C. This metabolite was measured here at a concentration of 1.1 ± 1.1 ng/mL (Table 1). In a separate study its concentration was determined to be 1.1 ± 0.9 ng/mL [10]. Quantification was against the internal standard [^2^H_7_]22 R-hydroxycholest-4-en-3-one, assuming a similar response factor to the authentic standards of the 7α,24 S- and 7α,25-dihydroxy acids. Based on this assumption the limit of quantification was 0.05 ng/mL. Analysis of CSF with and without cholesterol oxidase treatment revealed that the analogous trihydroxycholestenoic acid was essentially absent.

The MS^3^ ([M]^+^→[M-Py]^+^→) spectrum acquired at 4.16 min (Fig. 4F) reveals many similar fragment-ions to CA^4^-7α,24 S-diol-3-one (Fig. 4A and B) and CA^4^-7α,25-diol-3-one (Fig. 4C and D). The fragment-ion pattern of *m/z* 151.1 (*b_1_-12) and 179.1 (*b_3_-28) is characteristic of 7-hydroxy-3-oxo-4-ene sterols and is observed in each spectrum, however, fragment ions at *m/z* 209.1 (*b_3_+2) and 279.2 (*c_3_+2), observed in the spectrum of the unknown compound shown in Fig. 4F, are characteristic of 7,12-dihydroxy-3-oxo-4-ene sterols [14] (Supplementary Fig. 4). In the high *m/z* range of this spectrum, a distinctive fragment-ion of *m/z* 422.3 is evident. This is analogous to an abundant ion at *m/z* 392.3 characteristic of 7α,12α-dihydroxycholest-4-en-3-one (Supplemental Fig. 5) [14]. This fragment-ion corresponds to the neutral-loss of CO, NH and 2 × H_2_O (−79 Da) from the [M-Py]^+^ ion (Supplementary Fig. 4). Based on the evidence above, the unknown dihydroxyoxocholestenoic acid is presumptively identified as 7α,12α-dihydroxy-3-oxocholest-4-en-26-oic acid (CA^4^-7α,12α-diol-3-one). By generating a MRM chromatogram 585.4→501.3→422.3, CA^4^-7α,12α-diol-3-one is highlighted eluting at 4.16 and 4.64 min (Fig. 5D). This further supports the identification of CA^4^-7α,12α-diol-3-one as empirical data shows that B- and C-ring hydroxysterols usually elute later than side-chain hydroxysterols in our chromatographic system. Unpublished data from a study of plasma samples from patients with the peroxisomal disorder acyl-CoA oxidase 2 (ACOX2) deficiency (Fig. 1A) where cholestenoic acids, including 25 R- and unusually 25 S-epimers of CA^4^-7α,12α-diol-3-one build-up, substantiates the identification of the 25 R-epimer of CA^4^-7α,12α-diol-3-one in the current study eluting at 4.16 and 4.64 min.

7α,12α,25-Trihydroxycholest-4-ene-3,24-dione (C^4^-7α,12α,25-triol-3,24-dione, see Supplemental Fig. 6), an intermediate in the formation of bile acids from 25-hydroxycholesterol (C^5^-3β,25-diol) [15], is an isomer of CA^4^-7α,12α-diol-3-one. An authentic standard of this compound is not available and it is possible that it may give a similar MS^3^ spectrum to that assigned to CA^4^-7α,12α-diol-3-one and contribute to some of the ion-current at 4.17 and 4.62 min.

### 3.2 Dihydroxy-3-oxocholest-4-en-26-oic acids in CSF from CTX patients

CYP27A1 catalyses the (25 R)26-hydroxylation of cholesterol and further oxidation of the (25 R)26-hydroxy group to a (25 R)26-carboxyl group (Fig. 1A) [1,16]. Deficiency in CYP27A1, as observed in patients suffering from CTX, leads to reduced synthesis of cholestenoic acids and of the primary bile acid chenodeoxycholic acid [9,17,18].

#### 3.2.1 CA^4^-7α,24-diol-3-one, CA^4^-7α,25-diol-3-one, CA^4^-7α,12α-diol-3-one and CA^4^-7α,x-diol-3-one

**Fig. 6:**
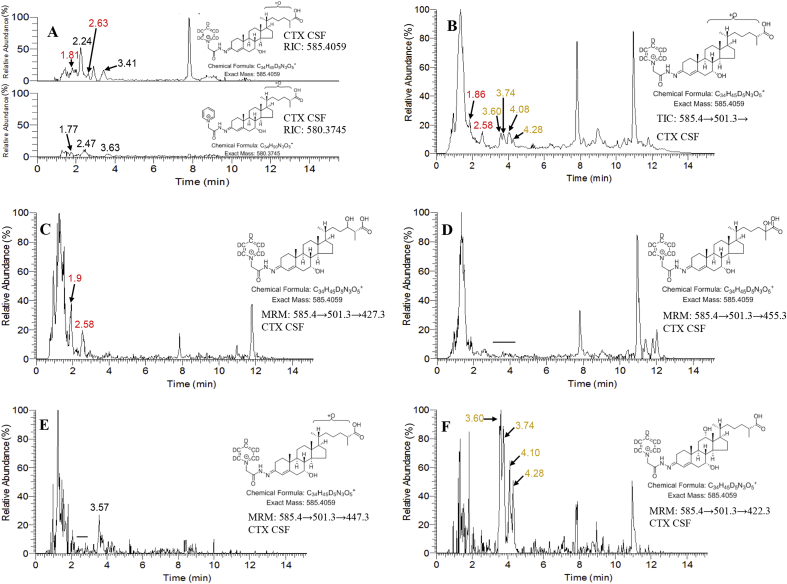
Analysis of CSF from a CTX patient. (A) LC-HRMS RICs (585.4059 ± 10 ppm, upper panel; 580.3745 ± 10 ppm, lower panel). The upper panel shows CTX CSF (SPE1-Fr1A) treated with cholesterol oxidase followed by [^2^H_5_]GP reagent, while the lower panel shows CTX CSF (SPE1-Fr1B) treated with [^2^H_0_]GP reagent in the absence of cholesterol oxidase. The RICs for [^2^H_5_]GP derivatised and [^2^H_0_]GP derivatised acids are plotted with the same y-axis. Data was acquired in the Orbitrap analyser. (B) LC-MS^3^ (585.4→501.3→) TIC appropriate to [^2^H_5_]GP derivatised dihydroxy-3-oxocholest-4-en-26-oic acids. LC-MS^3^ MRM transitions appropriate to (C) CA^4^-7α,24-diol-3-one, (D) CA^4^-7α,25-diol-3-one, (E) CA^4^-7α,x-diol-3-one and (F) CA^4^-7α,12α-diol-3-one. In (D) and (E) the horizontal bars indicate where the targeted acids are expected to elute. The MRMs in (C–F) were generated from data acquired in the LIT analyser of the Orbitrap Elite hybrid instrument with an *m/z* window of ±0.4. Chromatograms displayed in Fig. 5, Fig. 6 were recorded on different days and show a time shift of about 0.4 min for the later eluting peaks. Retention times were correlated by the analysis of QC samples within in each sample batch. Peaks are colour coded as in Fig. 3.

**Fig. 7:**
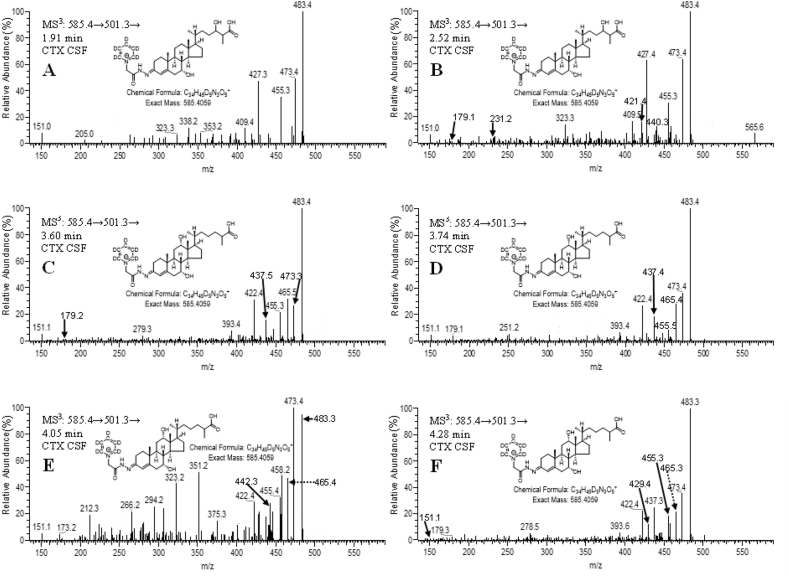
MS^3^ ([M]^+^→[M-Py]^+^→, i.e. 585.4→501.3→) spectra from CSF of a CTX patient. The spectra confirm the presence of dihydroxy-3-oxocholest-4-en-26-oic acids in CSF. Spectra shown in (A) & (B) are of *syn* and *anti* conformers of CA^4^-7α,24-diol-3-one, (C) & (E) are interpreted as *syn* and *anti* conformers of (25 S)CA^4^-7α,12α-diol-3-one, and (D) & (F) are interpreted as *syn* and *anti* conformers of (25 R)CA^4^-7α,12α-diol-3-one. Data was acquired in the LIT analyser of the Orbitrap Elite hybrid instrument with an accuracy of *m*/*z* ± 0.1 for most fragment-ions.

The LC-HRMS RICs of *m/z* 585.4059, corresponding to the [M]^+^ ion of dihydroxy-3-oxocholest-4-en-26-oic acids, recorded from CSF of CTX patients (n = 6) gave rise to a different profile of peaks to that observed normally in CSF (cf. Fig. 6A and Fig. 3C). Numerous peaks of unknown identity are evident in the chromatogram from the CTX samples. However, by generating a total ion chromatogram (TIC) for the LC-MS^3^ scan ([M]^+^→[M-Py]^+^→, i.e. 585.4→501.3→, Fig. 6B), which highlights GP-derivatised compounds via the neutral-loss of pyridine, peaks suggesting the presence of CA^4^-7α,24-diol-3-one (1.86 and 2.58 min) and of CA^4^-7α,12α-diol-3-one (3.74 and 4.28 min) were detected. Note, the chromatograms depicted in Fig. 3, Fig. 6 were recorded on different days, months apart, resulting in a shift in retention times. However, chromatographic peaks were correlated by recording QC samples with each batch of samples. Despite shifts in retention time between QC samples, the order of elution of monitored analytes did not change. Further evidence for these two acids was provided by generating RICs for the MRM transitions 585.4→501.3→427.3, which highlights CA^4^-7α,24-diol-3-one (Fig. 6C), and 585.4→501.3→422.3, which highlights CA^4^-7α,12α-diol-3-one (Fig. 6F). Confirmation of the presence of CA^4^-7α,24-diol-3-one in CSF from CTX patients was achieved by acquisition of MS^3^ spectra at 1.91 and 2.52 min (Fig. 7A and B) and comparison with the spectrum of the authentic standard (Fig. 4B). Despite the low intensity of the chromatographic peaks in Fig. 6, and hence MS^3^ spectra in Fig. 7, fragment-ions at *m/z* 151.1 and 179.1 characteristic of the 7-hydroxy-3-oxo-4-ene structure and *m/z* 427.3 characteristic of the 7,24-dihydroxy-3-oxocholest-4-en-26-oic acid are evident in the MS^3^ spectra of peaks eluting at 1.91 and 2.52 min (Fig. 7A and B). CA^4^-7α,24-diol-3-one was detected in each of the six CTX CSF samples analysed at a concentration of 0.1 ± 0.02 ng/mL but significantly lower (p < 0.01, Mann-Whitney test) than in control CSF (0.3 ± 0.1 ng/mL, Table 1).

The MS^3^ spectra of the chromatographic peaks at 3.74 and 4.28 min (Fig. 6F) give similar patterns of fragment-ions to CA^4^-7α,12α-diol-3-one present in normal CSF (cf. Fig. 7D and F with 4 F). In addition to fragment-ions at *m/z* 151.1 and 179.1 the spectra show a fragment-ion at *m/z* 422.3 characteristic of the 7,12-dihydroxy-3-oxocholest-4-en-26-oic acids. Interestingly, four chromatographic peaks were evident in Fig. 6F giving MS^3^ spectra attributable to CA^4^-7α,12α-diol-3-one (Fig. 7C–F). Normally only two chromatographic peaks are assigned to the CA^4^-7α,12α-diol-3-one structure (Fig. 3C). The four peaks in the CTX sample are suggestive of *syn* and *anti* conformers of the 25 R- and 25 S-epimers of CA^4^-7α,12α-diol-3-one. A similar pattern of peaks was found in plasma of patients suffering from ACOX2 deficiency (unpublished data), where both epimers are expected, substantiating the current identification.

The 25 S-epimer of CA^4^-7α,12α-diol-3-one, corresponding to the earlier eluting components in the twin pairs of peaks (3.60 and 4.10 min in Fig. 6F), was found in CTX patients at a level of 0.1 ± 0.1 ng/mL, but in normal plasma was not detected (Table 1). The 25 R-epimer was present in five of the six CTX patients studied (0.5 ± 0.6 ng/mL) and in the other patient neither epimer was detected. (25 R)CA^4^-7α,12α-diol-3-one was present at significantly lower levels in CTX CSF than in normal CSF (p < 0.05). The two epimers are interconvertible via the enzyme alpha-methylacyl-CoA racemase (AMACR) [1].

As mentioned above, C^4^-7α,12α,25-triol-3,24-dione, an intermediate in the formation of bile acids from C^5^-3β,25-diol [15], is an isomer of CA^4^-7α,12α-diol-3-one and may contribute some ion-current to the peaks assigned to (25 R)CA^4^-7α,12α-diol-3-one. However, C-25 is not asymmetric in C^4^-7α,12α,25-triol-3,24-dione (Supplemental Fig. 6) and will not give epimers at C-25 and cannot account for the peaks assigned to the (25 S)CA^4^-7α,12α-diol-3-one. Though at significantly lower level than in normal CSF, (25 R)CA^4^-7α,12α-diol-3-one was detected in CSF from CTX patients despite CYP27A1 deficiency. Notably, C^4^-7α,12α-diol-3-one is also elevated in CSF of CTX patients (data not shown) and could provide a substrate for carboxylation at C-26.

Despite generating the MRM chromatogram 585.4→501.3→455.3 to highlight CA^4^-7α,25-diol-3-one there was no evidence for this acid in CSF from CTX patients (Fig. 6D). The most abundant dihydroxy-3-oxocholest-4-en-26-oic acids in human CSF, CA^4^-7α,x-diol-3-one and (Fig. 3C) was not detected in CSF from CTX patients (Fig. 6E).

### 3.3 Dihydroxy-3-oxocholest-4-en-26-oic acids in CSF from SPG5 patients

Oxysterol 7α-hydroxylase (CYP7B1) catalyses the 7α-hydroxylation of most oxysterols [1,19,20]. Deficiency in this enzyme, as observed in patients suffering from SPG5, leads to elevated levels of C^5^-3β,25-diol, (25 R)26-hydroxycholesterol (C^5^-3β,26-diol) and CA^5^-3β-ol and reduced levels of 7α,25-dihydroxycholesterol (C^5^-3β,7α,25-triol), 7α,(25 R)26-dihydroxycholesterol (C^5^-3β,7α,26-triol) and CA^5^-3β,7α-diol, and their 3-oxo-4-ene equivalents in CSF [5,9]. Thus, dihydroxy-3-oxocholest-4-en-26-oic acids containing a 7α-hydroxy group are predicted to be reduced in CSF from SPG5 patients in comparison to healthy controls.

#### 3.3.1 CA^4^-7α,24-diol-3-one, CA^4^-7α,25-diol-3-one, CA^4^-7α,12α-diol-3-one and CA^4^-7α,x-diol-3-one

In 2014 we analysed CSF from SPG5 patients [9], but in the absence of authentic standards of CA^4^-7α,24-diol-3-one or CA^4^-7α,25-diol-3-one misidentified the former metabolite. Reassessment of our earlier data [9], now with access to authentic standards, allows definitive identification of CA^4^-7α,24-diol-3-one at a concentration of 0.1 ± 0.1 ng/mL and of CA^4^-7α,25-diol-3-one at 0.2 ± 0.3 ng/mL (n = 3) (Table 1).

Although authentic standards are still not available for CA^4^-7α,12α-diol-3-one, based on fragmentation data discussed above, we identify (25 R)CA^4^-7α,12α-diol-3-one in SPG5 CSF at a concentration of 0.6 ± 0.2 ng/mL and CA^4^-7α,x-diol-3-one at 0.6 ± 0.7 ng/mL, these values are considerably lower than the values for control CSF measured in this study (see Table 1). As in the case of control CSF, the concentrations of trihydroxycholest-5-en-26-oic acids were at most 20% of the analogous dihydroxycholest-4-en-26-oic acids (Supplemental Figs. 7A and B).

### 3.4 Dihydroxy-3-oxocholest-4-en-26-oic acids in control plasma

Control plasma (n = 24) was from a previously published study [10] and data from that study for dihydroxy-3-oxocholest-4-en-26-oic acids was compared to new data for CTX and SPG5 patients. CTX (n = 13) and SPG5 (n = 2) plasma was analysed in separate batches on different days to avoid cross contamination from abundant metabolites. However, in each sequence a QC sample was included in order to calibrate retention times to those in control plasma.

#### 3.4.1 CA^4^-7α,24-diol-3-one

**Fig. 8:**
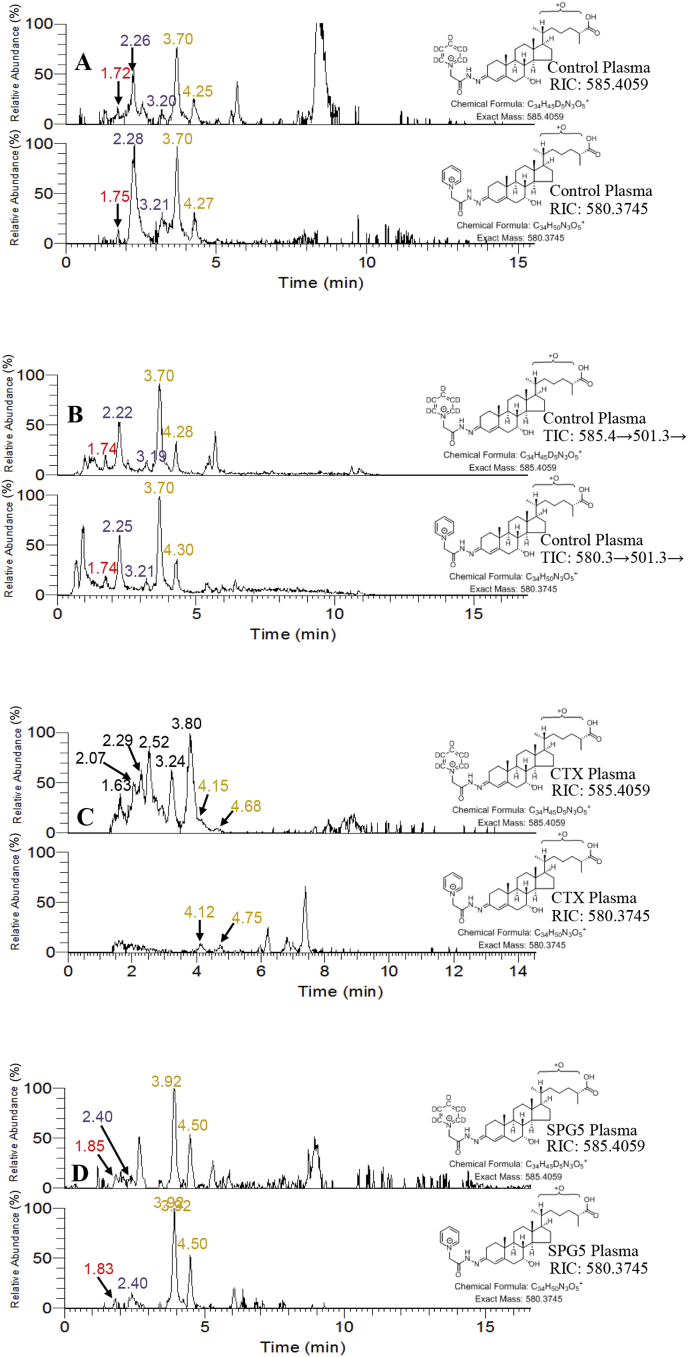
LC-HRMS RICs ±10 ppm appropriate to the analysis of [^2^H_5_]GP and [^2^H_0_]GP derivatised dihydroxy-3-oxocholest-4-en-26-oic acids in control (A), CTX (C) and SPG5 (D) plasma. The RICs for [^2^H_5_]GP derivatised dihydroxy-3-oxocholest-4-en-26-oic acids are plotted on the same y-axis scale as the RICs for [^2^H_0_]GP derivatised acids. (B) LC-MS^3^ TICs appropriate to [^2^H_5_]GP and [^2^H_0_]GP derivatised dihydroxy-3-oxocholest-4-en-26-oic acids in control plasma. Control, CTX and SPG5 plasma were analysed on different days which causes a small deviation in retention time. Peaks were correlated by recording QCs with each sample batch. HRMS data was recorded in the Orbitrap and MS^3^ data in the LIT of the Orbitrap Elite hybrid instrument. Peaks are colour coded as in Fig. 3.

In plasma collected from healthy controls (n = 24), CA^4^-7α,24-diol-3-one was reported to be present at a concentration of 0.05 ± 0.04 ng/mL (Table 2, Fig. 8A) [10]. CA^4^-7α,24-diol-3-one was more abundant than its 3β-hydroxy-5-ene analogue, CA^5^-3β,7α,24-triol, by about 10-fold. Although present at low levels in plasma (Fig. 8A and B), the identification of CA^4^-7α,24-diol-3-one was confirmed by an acquisition of an MS^3^ spectrum at 1.74 min as shown Supplemental Fig. 8A.

**Table 2:** Concentrations of dihydroxy-3-oxocholest-4-en-26-oic acids in plasma.

Compound	Control plasma^1^, n = 24			CTX plasma, n = 13				SPG5 plasma	
	Mean	Median	SD	Mean	Median	SD	Significance^2^	P1	P2
	ng/mL	ng/mL	ng/mL	ng/mL	ng/mL	ng/mL	CTX v Control	ng/mL	ng/mL
CA^4^-7α,24-diol-3-one	0.05	0.04	0.04	0.15	0.06	0.19	P > 0.05	0.23	0.15
CA^4^-7α,x-diol-3-one	0.92	0.89	0.30	ND	ND	NA	P < 0.01	0.33	0.20
CA^4^-7α,25-diol-3-one	ND	ND	NA	ND	ND	NA	NA	ND	ND
(25 R)CA^4^-7α,12α-diol-3-one	1.56	1.38	1.24	0.29	0.03	0.43	P < 0.01	1.91	0.78
(25 S)CA^4^-7α,12α-diol-3-one	ND	ND	NA	0.08	0.03	0.12	P < 0.01	ND	ND

Quantification was made utilising LC-HRMS RICs with [^2^H_7_]22 R-hydroxycholest-4-en-3-one as the internal standard.

1.Data from Abdel-Khalik et al. [10].

2.Mann-Whitney test.

#### 3.4.2 CA^4^-7α,25-diol-3-one

CA^4^-7α,25-diol-3-one was determined to be the second most abundant dihydroxy-3-oxocholest-4-en-26-oic acid in CSF, but was only detected at trace levels in plasma (<0.05 ng/mL).

#### 3.4.3 CA^4^-7α,x-diol-3-one

The LC-HRMS RIC for the [M]^+^ ion at *m/z* 585.4059 revealed peaks at 2.26 and 3.20 min assigned to the *syn* and *anti* conformers of CA^4^-7α,x-diol-3-one (Fig. 8A). The identification was confirmed by acquisition of MS^3^ spectra (Supplemental Figs. 8B and C). Fragment-ions at *m/z* 151.1, 179.1 and 231.1 characteristic of the 7-hydroxy-3-oxo-4-ene structure were evident. In the high *m/z* range fragment ions at *m/z* 419.3, 440.3 and 447.3 were observed as in the spectra recorded in CSF and annotated as CA^4^-7α,x-diol-3-one (Fig. 4E). In control plasma, CA^4^-7α,x-diol-3-one was determined to be present at a concentration of 0.92 ± 0.30 ng/mL (Table 2), more than ten-fold greater than the 3β-hydroxy-5-ene analogue, CA^5^-3β,7α,x-triol [10].

#### 3.4.4 CA^4^-7α,12α-diol-3-one

(25 R)CA^4^-7α,12α-diol-3-one is the most abundant dihydroxy-3-oxocholest-4-en-26-oic acid in plasma [10]. *Syn* and *anti* conformers of the 25 R-epimer were assigned to peaks at 3.70 and 4.25 min in the LC-HRMS RIC (Fig. 8A). MS^3^ spectra recorded at 3.70 and 4.28 min confirmed these identifications (Supplemental Figs. 8D and E). Fragment-ions at *m/z* 151.1, 179.1, 209.1 and 279.2 m*/z* were observed, as in the spectra assigned to CA^4^-7α,12α-diol-3-one in CSF (Fig. 4F). In the high *m/z* range the fragment-ion at *m/z* 422.3 was present which also agrees with the spectrum recorded for CA^4^-7α,12α-diol-3-one in CSF. Plasma concentrations of 1.56 ± 1.24 ng/mL were determined for CA^4^-7α,12α-diol-3-one in control samples (Table 2), the 3β-hydroxy-5-ene analogue was at least ten-fold less abundant. There was no evidence for the 25 S-epimer in the control plasma samples analysed.

### 3.5 Dihydroxy-3-oxocholest-4-en-26-oic acids in plasma from CTX patients

Plasma was analysed from 13 CTX patients.

#### 3.5.1 CA^4^-7α,24-diol-3-one, CA^4^-7α,25-diol-3-one and CA^4^-7α,x-diol-3-one

CA^4^-7α,24-diol-3-one was detected in CTX plasma at about the limit of detection of the method (0.05 ng/mL) in 11 of the 13 samples analysed, in one other it was absent but was present at 0.35 ng/mL in the other sample (Table 2). There was little evidence for the presence of CA^4^-7α,25-diol-3-one or CA^4^-7α,x-diol-3-one in plasma from CTX patients. The absence of the two acids in plasma from CTX patients agrees with the inability to detect these dihydroxy-3-oxocholest-4-en-26-oic acids in CSF from CTX patients.

#### 3.5.2 CA^4^-7α,12α-diol-3-one

The RIC for *m/z* 585.4059 (Fig. 8C) gave two peaks at 4.15 and 4.68 min and the MRM chromatogram of 585.4→501.3→422.3 (Supplemental Fig. 9) at 4.10 and 4.70 min which are assigned to (25 R)CA^4^-7α,12α-diol-3-one based on comparison of retention time to a QC sample run in the same sample batch. The mean concentration of (25 R)CA^4^-7α,12α-diol-3-one in CTX plasma (0.29 ± 0.43 ng/mL) was significantly lower than in healthy controls (1.56 ± 1.24 p < 0.01, Table 2), and of the 13 CTX samples analysed the acid was only identified in 8 samples. In 7 samples there was a suggestion of the presence of the (25 S)-epimer but only at trace levels (0.08 ± 0.12 ng/mL).

### 3.6 Dihydroxy-3-oxocholest-4-en-26-oic acids in plasma from SPG5 patients

As was the case with CSF, plasma from SPG5 patients was analysed for dihydroxy-3-oxocholest-4-en-26-oic acids in a study performed by us in 2014 [9], but in the absence of authentic standards. In the current study we have analysed plasma from a further two patient samples.

#### 3.6.1 CA^4^-7α,24-diol-3-one and CA^4^-7α,25-diol-3-one

A peak corresponding to CA^4^-7α,24-diol-3-one was observed at 1.85 min in the LC-HRMS RIC for *m/z* 585.4059 (Fig. 8D) and at 1.82 min the MRM chromatogram 585.4→501.3→427.3 (Supplemental Fig. 10A) from analysis of SPG5 plasma, the retention time being almost identical to that recorded for CA^4^-7α,24-diol-3-one in a QC plasma sample run within in the same batch of samples (Supplemental Fig. 10B). The plasma levels of CA^4^-7α,24-diol-3-one in the two SPG5 patients analysed here were 0.15 and 0.23 ng/mL, somewhat higher than levels seen in healthy controls (0.05 ± 0.04 ng/mL, Table 2). There was no convincing evidence for the presence of CA^4^-7α,25-diol-3-one in SPG5 plasma.

#### 3.6.2 CA^4^-7α,12α-diol-3-one

The LC-HRMS RIC for *m/z* 585.4059 (Fig. 8D) and MRM chromatogram 585.4→501.3→422.3 (Supplemental Fig. 10D) revealed two peaks at 3.92 and 4.50 min assigned to the *syn* and *anti* conformers of (25 R)CA^4^-7α,12α-diol-3-one. The concentrations of (25 R)CA^4^-7α,12α-diol-3-one in the two SPG5 patients (1.91 and 0.78 ng/mL) were at similar levels to healthy controls, i.e. 1.56 ± 1.24 ng/mL (Table 2). No (25 S)CA^4^-7α,12α-diol-3-one was detected.

#### 3.6.3 CA^4^-7α,x-diol-3-one

In the LC-HRMS RIC for *m/z* 585.4059 (Fig. 8D) and the MRM chromatogram 585.4→501.3→447.3 (Supplemental Fig. 10C) a peak at 2.40 min is evident which gives an MS^3^ spectrum (Supplemental Fig. 8F) similar to that assigned to CA^4^-7α,x-diol-3-one in CSF (Fig. 4E) and control plasma (Supplemental Fig. 8B). In the low *m/z* range of the MS^3^ spectrum acquired from the SPG5 patient, fragment-ions at *m/z* 151.1, 179.1 and 231.1 were detected while fragment ions *m/z* 419.3, 440.3 and 447.3 were detected in the high *m/z* range. CA^4^-7α,x-diol-3-one was determined to be present in plasma from SPG5 patients at concentrations of 0.33 and 0.2 ng/mL, lower than observed in any of the 24 healthy controls (range 0.4–1.33 ng/ml, mean ± SD 0.92 ± 0.30).

## 4 Discussion

Dihydroxy-3-oxocholest-4-en-26-oic acids are present in human CSF and plasma [3,9,10]. These consist of CA^4^-7α,24-diol-3-one, CA^4^-7α,25-diol-3-one, CA^4^-7α,12α-diol-3-one and a fourth acid CA^4^-7α,x-diol-3-one where the location of the second hydroxy group is probably on the C_17_ side-chain. The concentrations of these dihydroxy-3-oxocholest-4-en-26-oic acids in CSF follow the order CA^4^-7α,x-diol-3-one > CA^4^-7α,25-diol-3-one > (25 R)CA^4^-7α,12α-diol-3-one > CA^4^-7α,24-diol-3-one (Table 1). In plasma from healthy controls the order is (25 R)CA^4^-7α,12α-diol-3-one > CA^4^-7α,x-diol-3-one > CA^4^-7α,24-diol-3-one (Table 2) [10]. It is noteworthy that the concentrations of CA^4^-7α,24-diol-3-one, CA^4^-7α,25-diol-3-one and CA^4^-7α,x-diol-3-one are higher in CSF than plasma, suggesting that they are formed, at least in part, in the central nervous system (CNS). CA^4^-7α,24-diol-3-one could be formed via two different pathways, (i) via the acidic pathway of bile acid biosynthesis as depicted in Fig. 1A or (ii) via a pathway starting with 24 S-hydroxylation of cholesterol by CYP46A1 (Fig. 1B) [21]. In fact, all of the enzymes, or their transcripts [3], including AMACR [22], required by the acidic pathway for biosynthesis of CA^4^-7α,24-diol-3-one are expressed in brain. The latter steps of the acidic pathway leading to the formation the diastereomers (25 R)CA^4^-7α,24 S-diol-3-one, (25 R)CA^4^-7α,24 R-diol-3-one, (25 S)CA^4^-7α,24 S-diol-3-one and (25 S)CA^4^-7α,24 R-diol-3-one are shown in Fig. 1C [23]. The arm of the pathway catalysed by D-bifunctional protein (DBP) is dominant in the liver leading to the formation of the (25 R)CA^4^-7α,24 R-diol-3-one [2], however, the relative importance of this and the other arm catalysed by L-bifunctional protein (LBP) leading to (25 S)CA^4^-7α,24 S-diol-3-one or (25 R)CA^4^-7α,24 S-diol-3-one in the CNS is not known. Alternatively, a pathway starting with 24 S-hydroxylation of cholesterol by CYP46A1, which is highly expressed in brain [1], can lead to the biosynthesis of both (25 R)CA^4^-7α,24 S-diol-3-one and also (25 S)CA^4^-7α,24 S-diol-3-one (Fig. 1B). The second step in this pathway, 7α-hydroxylation of 24 S-hydroxycholesterol (C^5^-3β,24 S-diol), is catalysed by CYP39A1, which is only weakly expressed in brain [22]. However, another route could be via (25 R)26-hydroxylation by CYP27A1 or CYP46A1 [24] and subsequent (25 R)26-carboxylation (Fig. 1B), before or after 7α-hydroxylation via CYP7B1, which is itself is highly expressed in brain [20]. C^5^-3β,24 S-diol is a known substrate of CYP27A1 [25]. Still yet another route for the formation of CA^4^-7α,24 S-diol-3-one could start with 24 S-hydroxylation of 7α-hydroxycholesterol (C^5^-3β,7α-diol) by CYP46A1 [24]. C^5^-3β,7α-diol is not synthesised in brain but may be imported from the periphery.

In the absence of additional authentic standards we cannot be sure of the structure of CA^4^-7α,x-diol-3-one, but the MS^3^ fragmentation pattern suggests that the second hydroxy group is located on C-22 or C-23. CYP3A4 has 23 R-hydroxylase activity [26], and is expressed in human brain [27].

Surprisingly, in both CSF and plasma from CTX patients CA^4^-7α,24-diol-3-one and CA^4^-7α,12α-diol-3-one were detected, the latter acid as both 25 S and 25 R-epimers. Shown in Fig. 1B is a potential pathway for the formation of CA^4^-7α,12α-diol-3-one in the absence of CYP27A1. CYP3A4 has been shown to have (25 S)26-hydroxylase activity [26] and we speculate that the primary alcohol formed could be oxidised further to a carboxylic acid by this enzyme. In studies with recombinant human enzyme we have confirmed that CYP3A4 has (25 S)26-hydroxylase activity towards C^5^-3β,7α-diol (unpublished data). Mast et al. noted that recombinant CYP46A1 could further hydroxylate C^5^-3β,24 S-diol to 24 S,26-dihydroxycholesterol (C^5^-3β,24 S,26-triol) [24], we speculate that the enzyme may also oxidise C-26 to a carboxylic acid, which could be metabolised further to 25 R- and 25 S-epimers of CA^4^-7α,24 S-diol-3-one (Fig. 1B). CA^4^-7α,x-diol-3-one could not be detected in CSF or plasma from CTX patients.

Analysis of CSF (Table 1) and plasma (Table 2) from SPG5 patients revealed that CA^4^-7α,24-diol-3-one, CA^4^-7α,25-diol-3-one, (25 R)CA^4^-7α,12α-diol-3-one and CA^4^-7α,x-diol-3-one are formed even in the absence of CYP7B1 activity, with 7α-hydroxylation presumably being catalysed by CYP39A1 or CYP7A1 [28,29]. In CSF, the concentrations of each of the four acids is reduced in SPG5 patients, although not to statistical significance in the case of (25 R)CA^4^-7α,12α-diol-3-one, indicating that CYP7B1 has a role in their biosynthesis. In contrast to control samples where the concentrations of CA^4^-7α,24-diol-3-one, CA^4^-7α,25-diol-3-one and CA^4^-7α,x-diol-3-one are higher in CSF than plasma, the concentration of CA^4^-7α,24-diol-3-one is higher in SPG5 plasma than CSF. This suggests that CYP7B1 is important for the biosynthesis of CA^4^-7α,24-diol-3-one in brain.

The biological relevance of the dihydroxy-3-oxocholest-4-en-26-oic acids identified in this work is unknown. While cholestenoic acids with a 3β-hydroxy-5-ene structure are ligands to LXRs, sterols with a 3-oxo-4-ene group do not appear to activate these receptors.

Although we have only limited clinical data for the CTX and SPG5 patients studied in this work the levels of the dihydroxy-3-oxocholest-4-en-26-oic acids determined in CSF do provide confirmatory diagnostic biomarkers of the two diseases. Whether or not the levels of these acids provide an indication of the severity of disease requires further study.
