# Supplementary material for: Identification of 7α,24-dihydroxy-3-oxocholest-4-en-26-oic and 7α,25-dihydroxy-3-oxocholest-4-en-26-oic acids in human cerebrospinal fluid and plasma
Source: Biochimie. 2018 Oct;153:86–98. doi: 10.1016/j.biochi.2018.06.020 (PMC6171785; doi:10.1016/j.biochi.2018.06.020)
Supplement: Multimedia component 1 [file mmc1.pdf]

S1

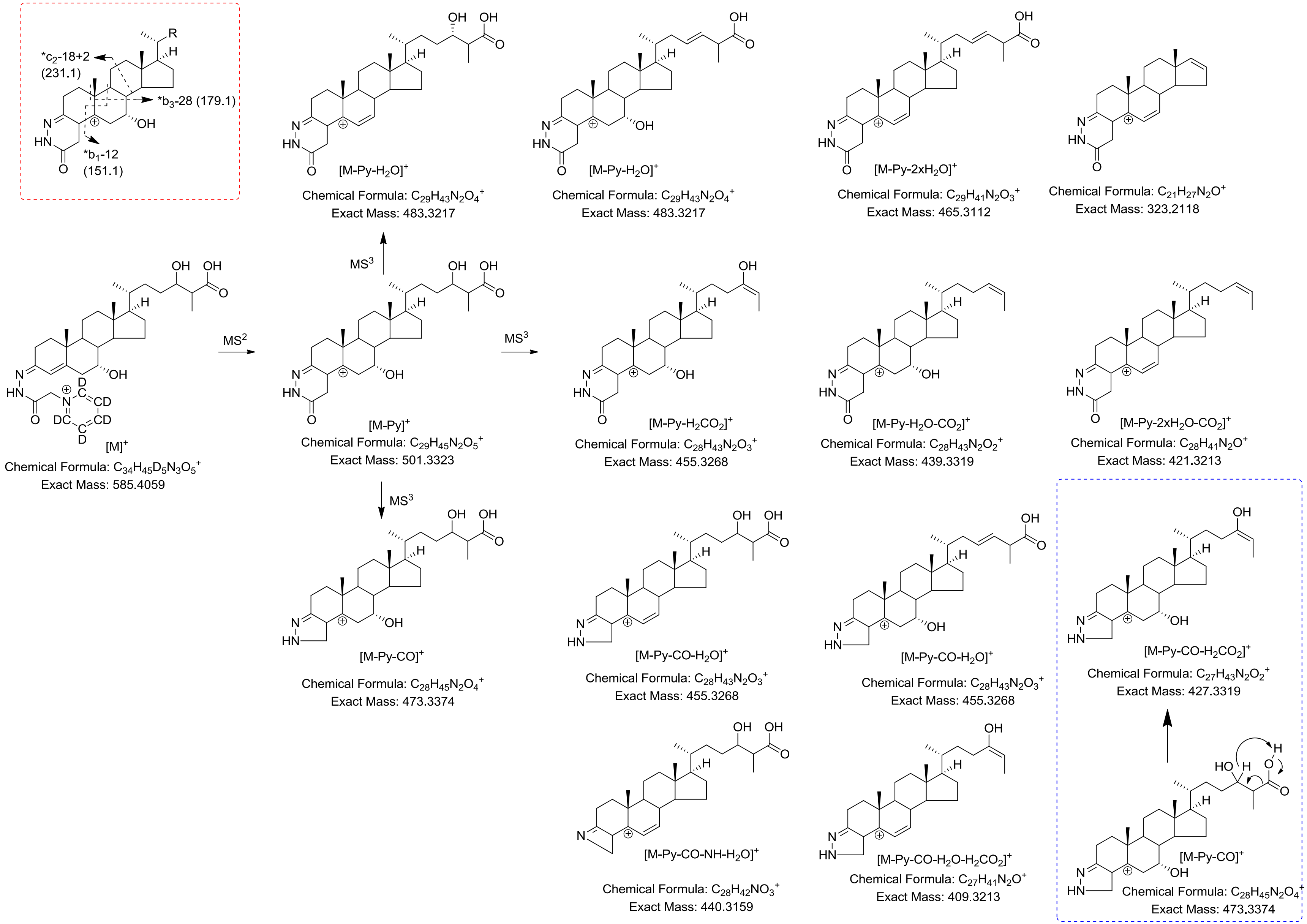


Figure S1. Suggested fragmentation of GP-derivatise CA$^4$-7α,24-diol-3-one. Shown in the blue box is the CRF mechanism proposed to give the characteristic fragment-ion at *m/z* 427.3. The inset in the red box shows cleavage in the steroid ring system. Py = pyridine.

S2

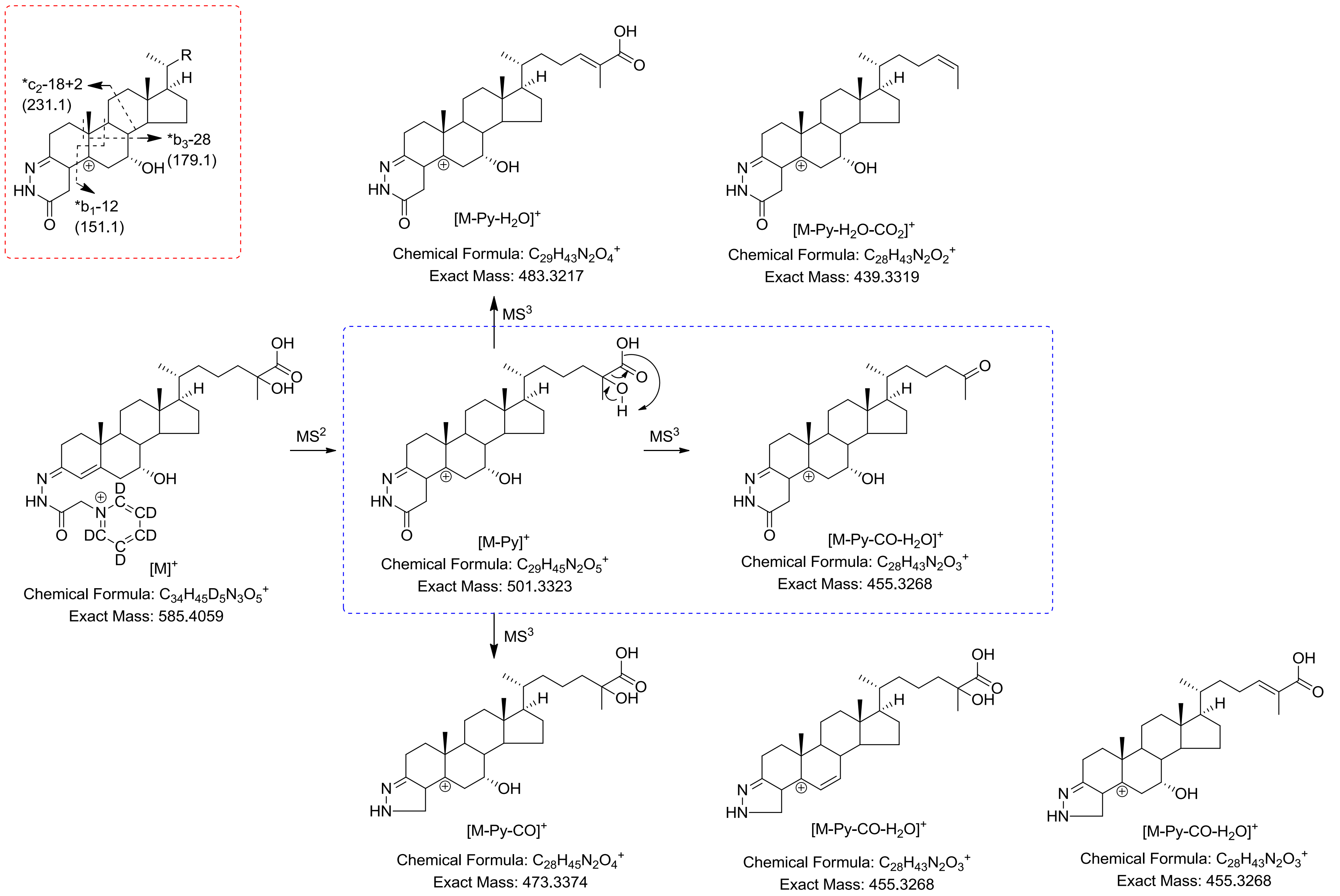


Figure S2. Suggested fragmentation of GP-derivatise CA[4]-7α,25-diol-3-one. Shown in the blue box is the CRF mechanism proposed to give the abundant fragment-ion at *m/z* 455.3. The inset in the red box shows cleavage in the steroid ring system. Py = pyridine.

# S3

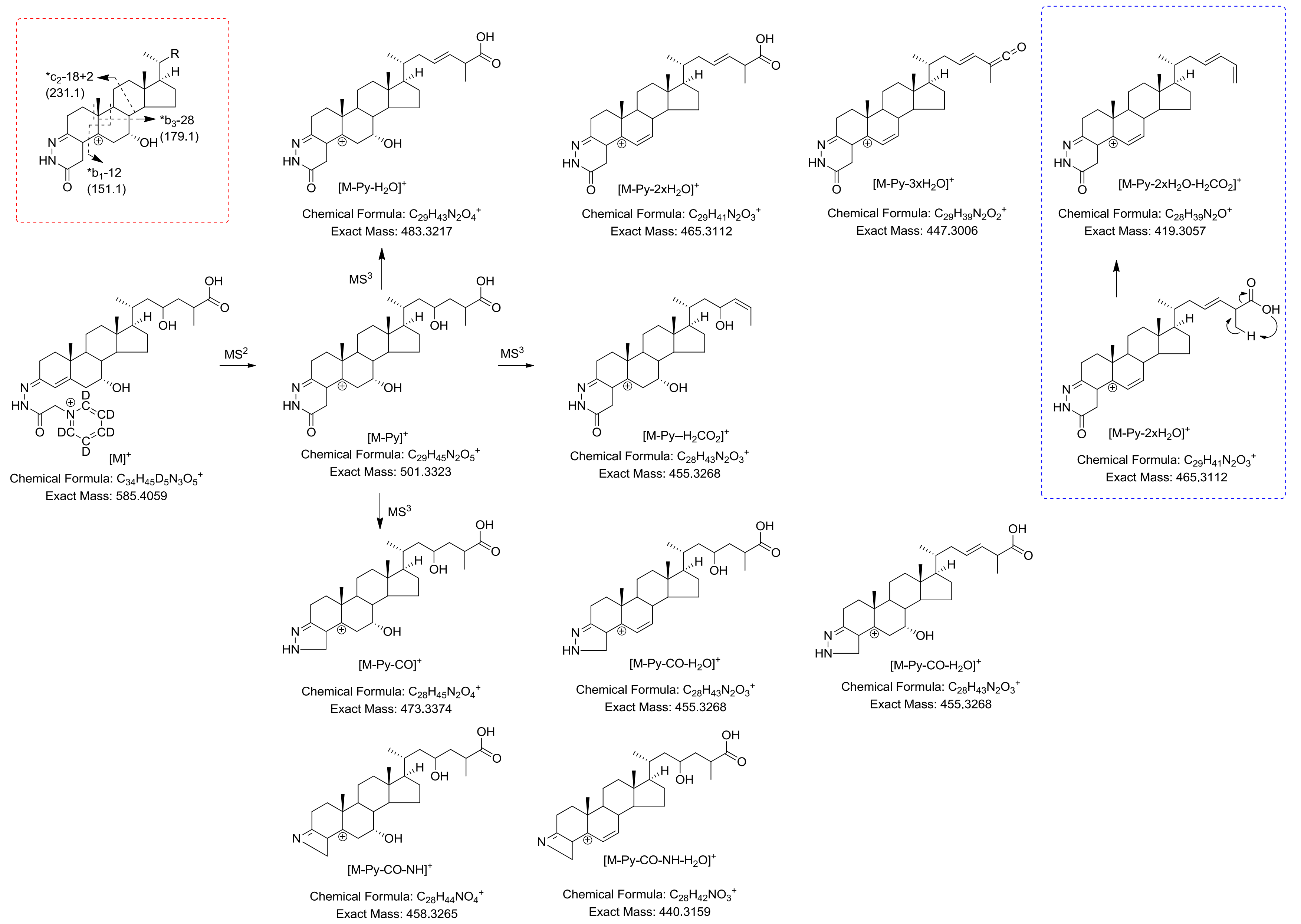


Figure S3. Suggested fragmentation of GP-derivatise CA$^4$-7α,x-diol-3-one. The second hydroxy group is drawn at C-23. Shown in the blue box is the CRF mechanism proposed to give the characteristic fragment-ion at *m/z* 419.3. The inset in the red box shows cleavage in the steroid ring system. Py = pyridine.

**S4**

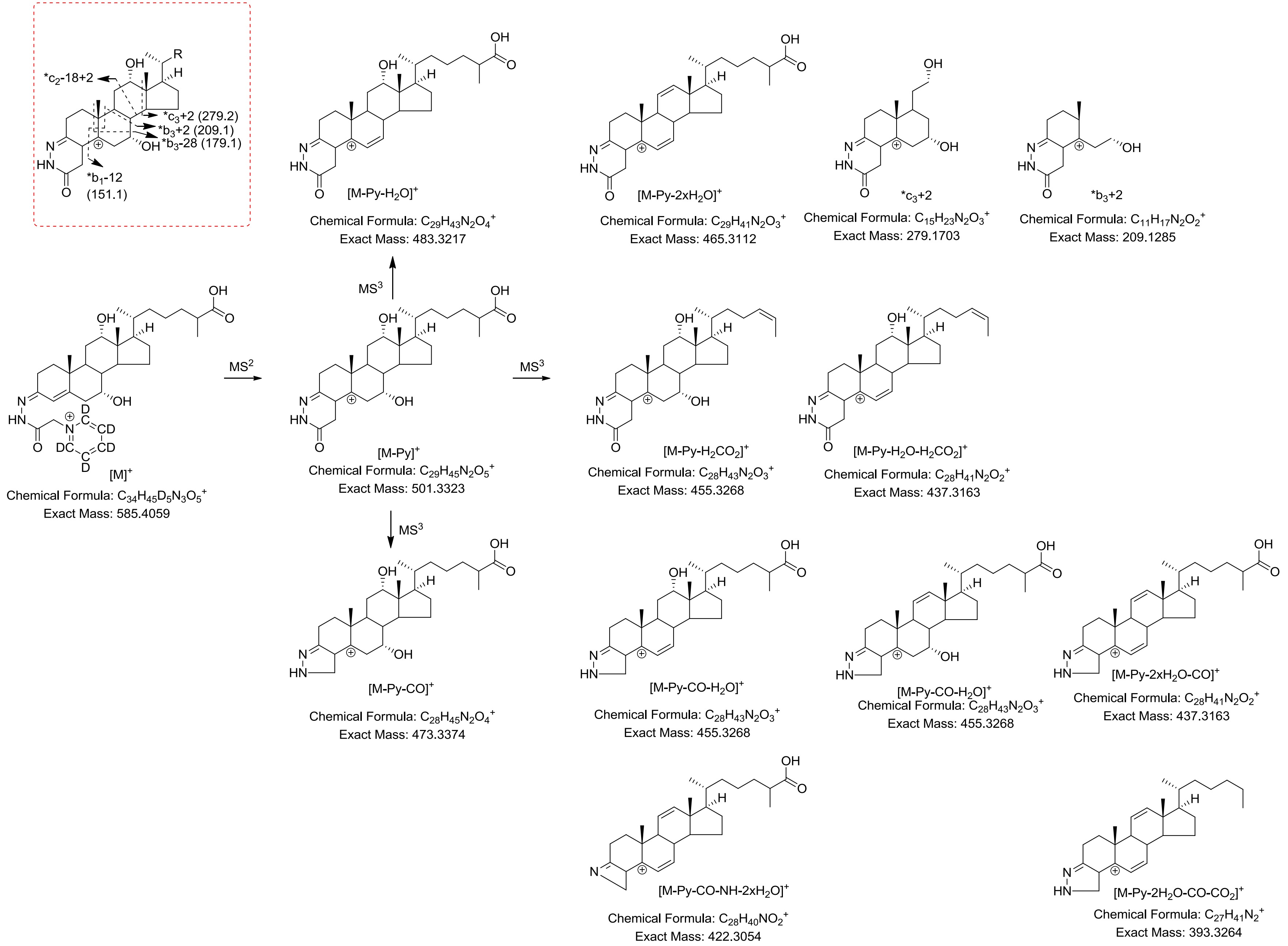


Figure S4. Suggested fragmentation of GP-derivatise CA[4]-7α,12α-diol-3-one. The inset in the red box shows cleavage in the steroid ring system. Py = pyridine.

# S5

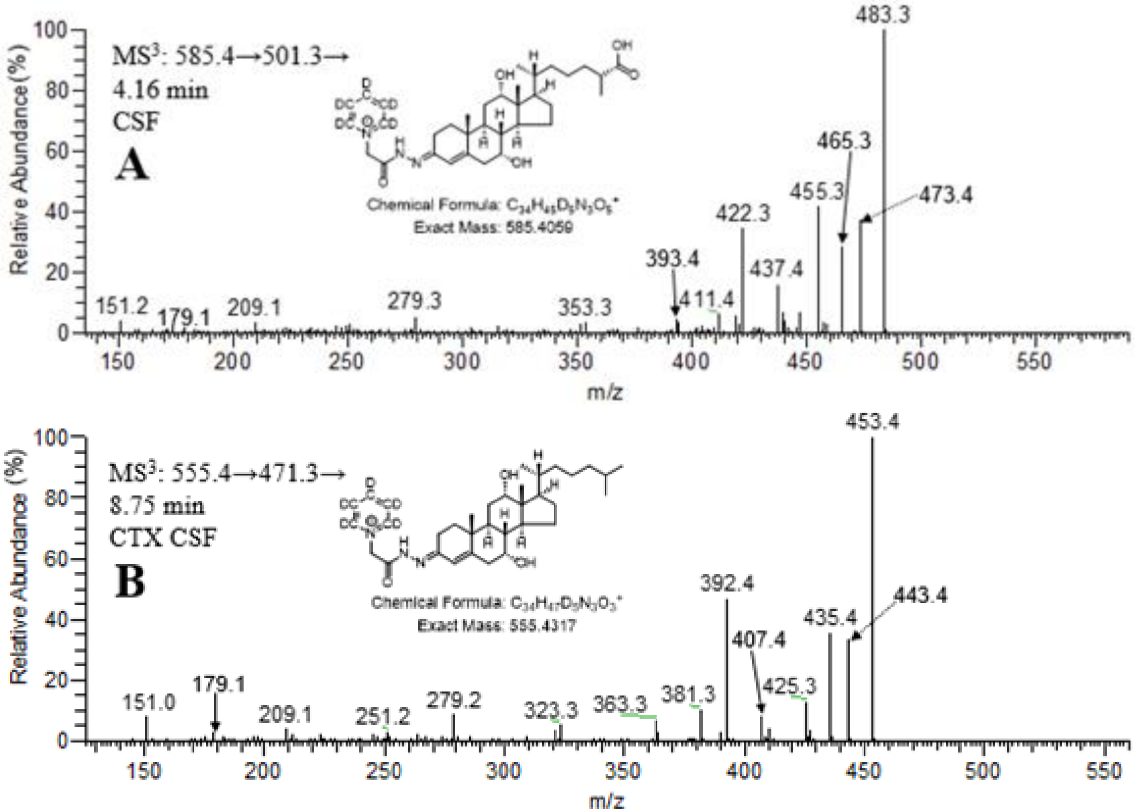


Figure S5. Comparison of the MS³ ([M]⁺→[M-Py]⁺→) spectra of (A) CA⁴-7α,12α-diol-3-one and (B) C⁴-7α,12α-diol-3-one. Both spectra show the steroid-ring fragment ions at *m/z* 151.1, 179.1,209.1 and 279.2. The fragment-ion at *m/z* 422.3 in (A) is displaced by 30 Da in (B), both ions correspond to [M-Py-CO-NH-2x$H_2O$]$^+$. Py = pyridine. Data was acquired in the LIT analyser of the Orbitrap Elite hybrid instrument with an accuracy of *m/z* ± 0.1 for most fragment-ions.

# S6

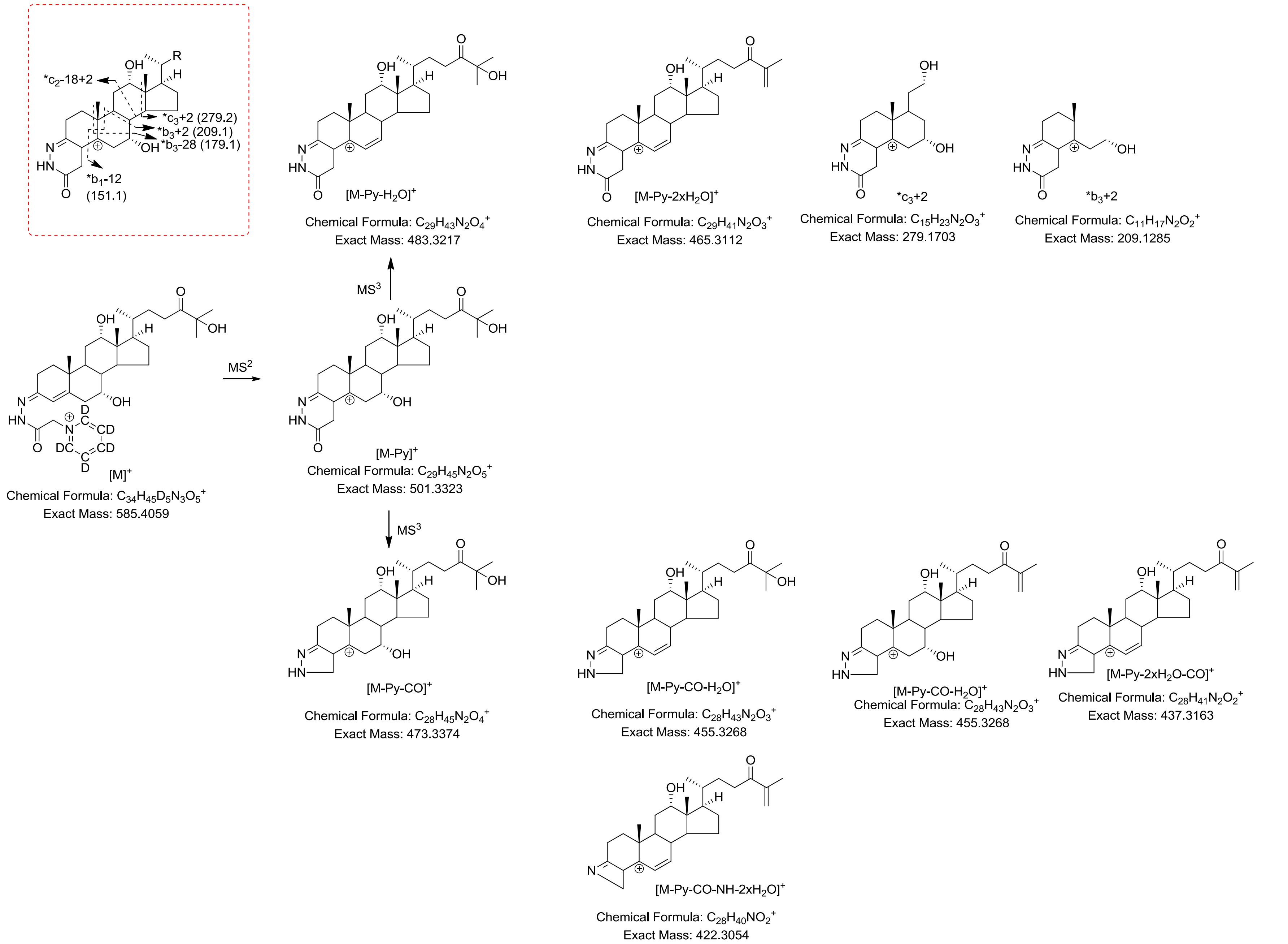


Figure S6. Suggested fragmentation of GP-derivatise C[4]-7α,12α,25-triol-3,24-dione. The inset in the red box shows cleavage in the steroid ring system. Py = pyridine.

S7

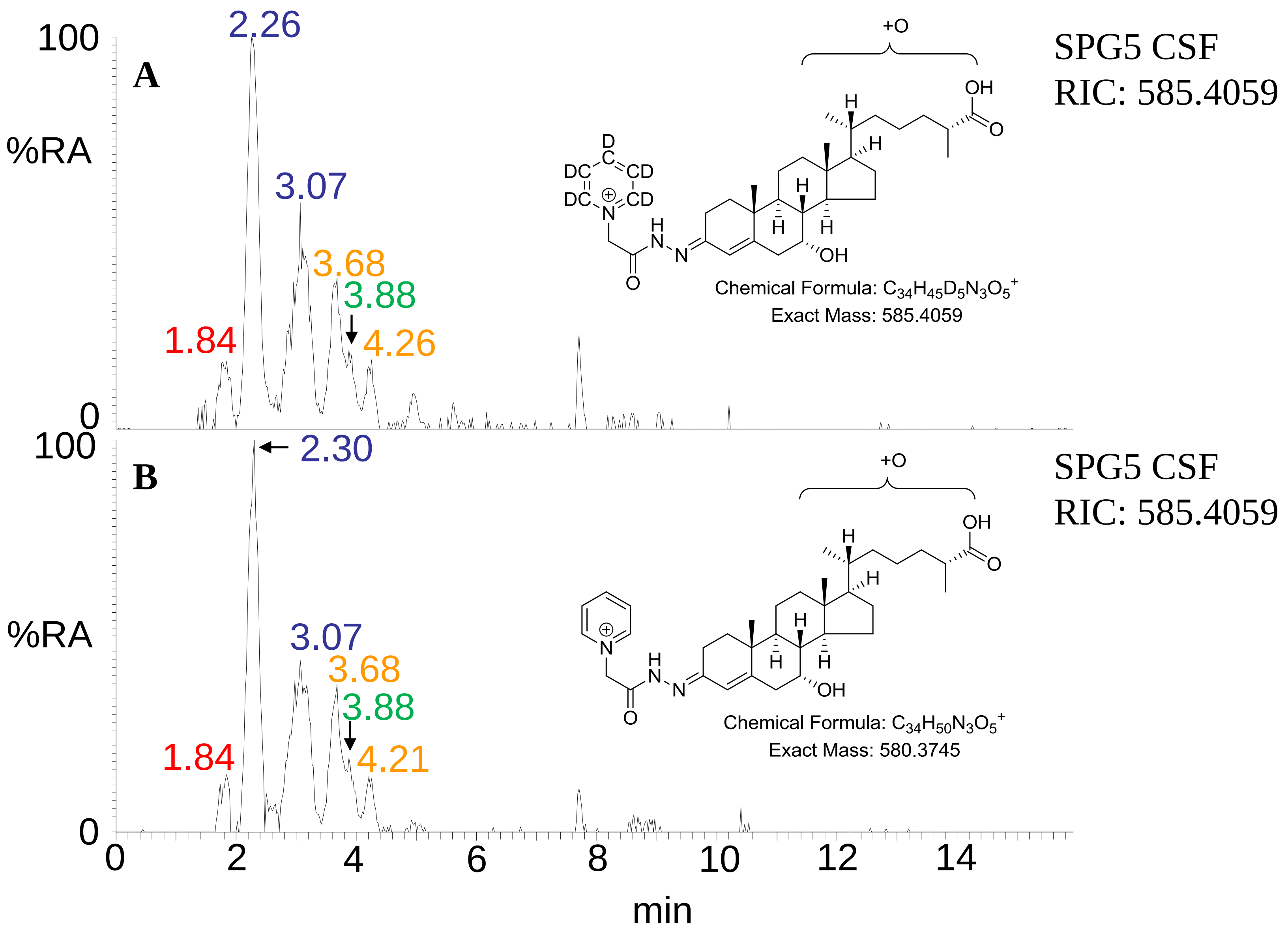


Figure S7A & B. RICs ± 10 ppm from a CSF sample of an SPG5 patient (A) treated with cholesterol oxidase and [$^{2}H_{5}$]GP and (B) treated with [$^{2}H_{0}$]GP in the absence of cholesterol oxidase. The RICs correspond to [M]$^{+}$ ions of GP-derivatiseed dihydroxy-3-oxocholest-4-en-26-oic acids.

**S7**

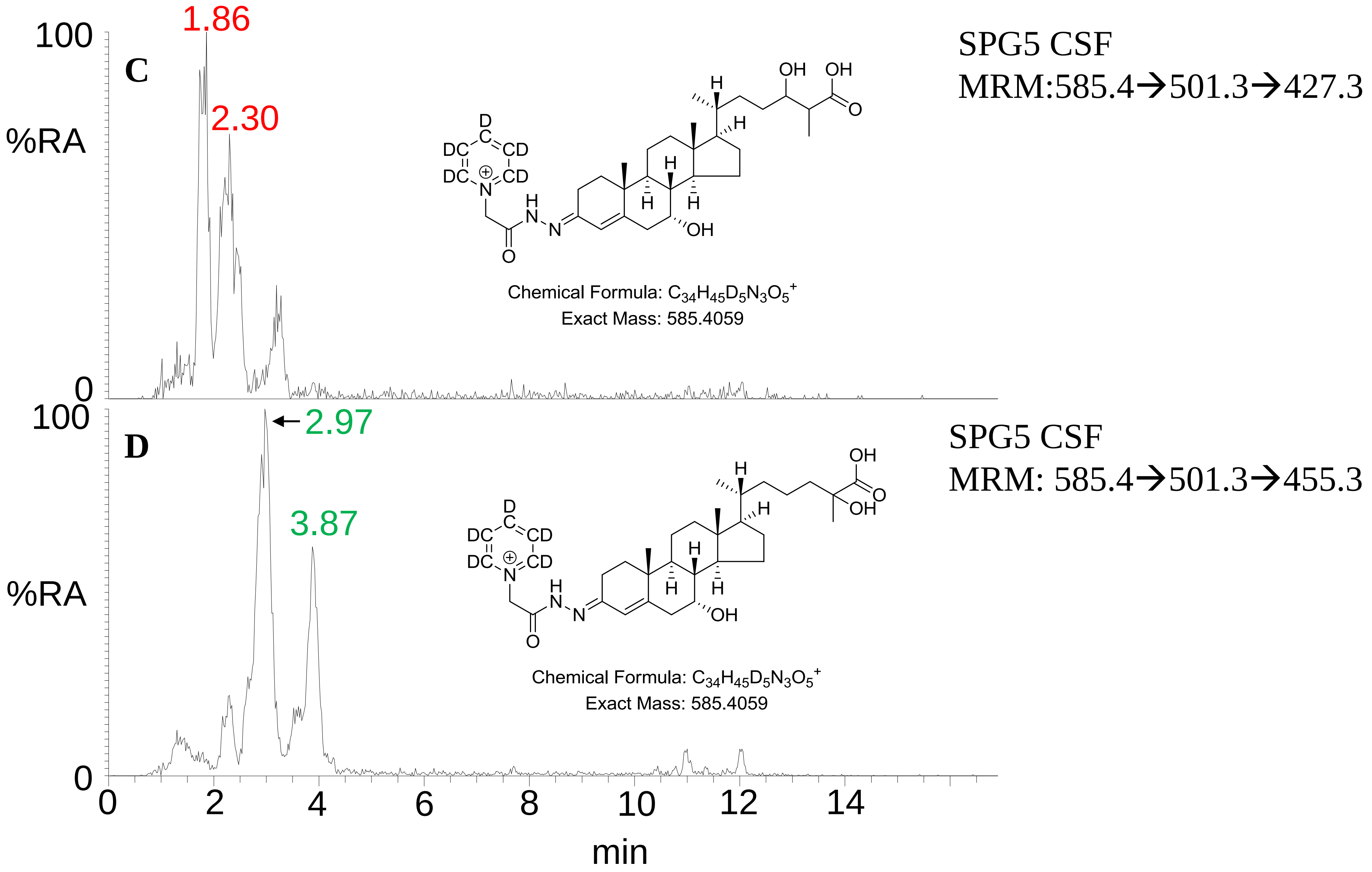


Figure S7C & D. MRM chromatograms from a CSF sample of an SPG5 patient to highlight (C) $CA^{4}$-7α,24-diol-3-one and (D) $CA^{4}$-7α,25-diol-3-one.

S7

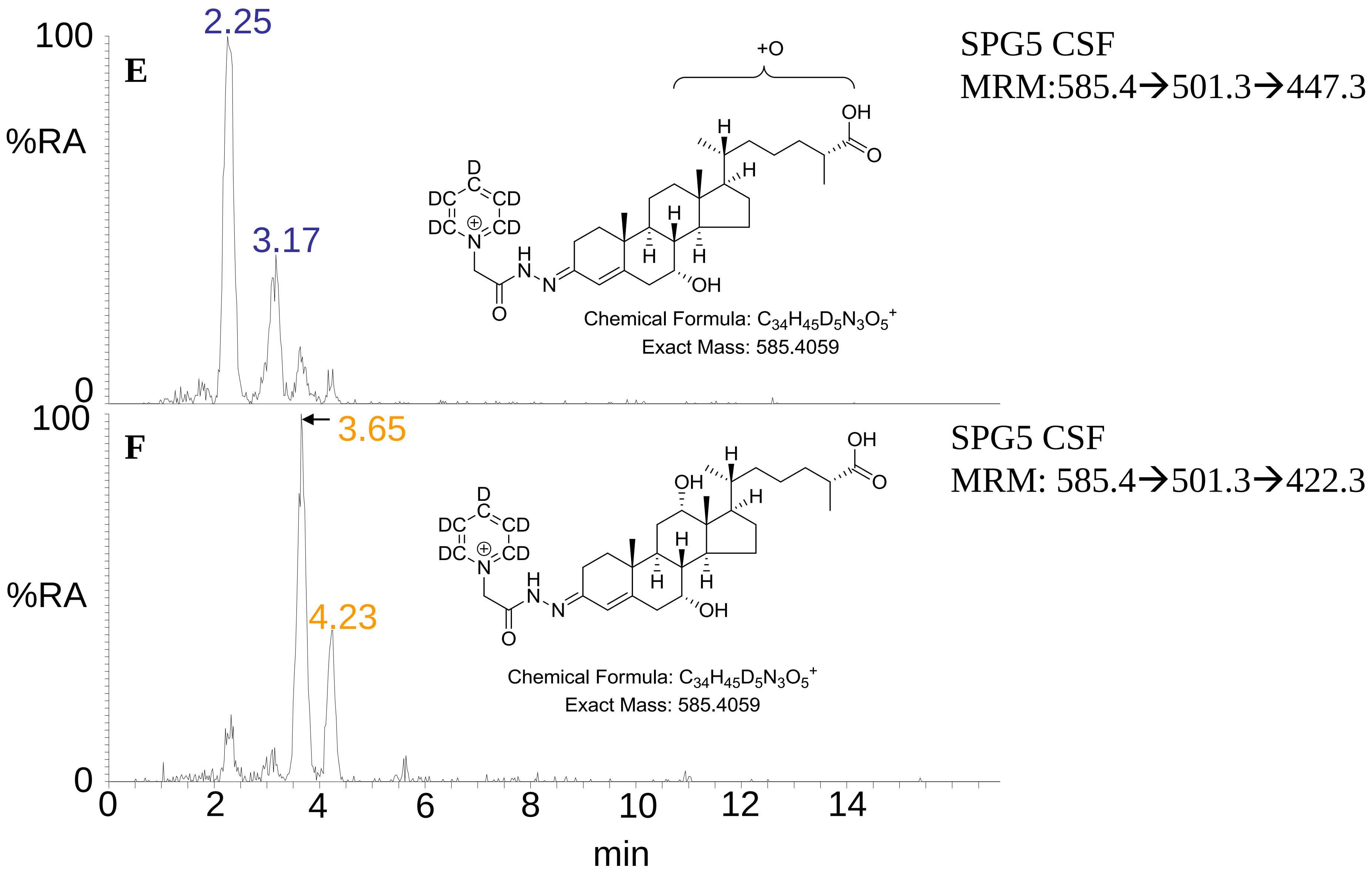


Figure S7E & F. MRM chromatograms from a CSF sample of an SPG5 patient to highlight (E) CA[4]-7α,x-diol-3-one and (F) CA[4]-7α,12α-diol-3-one .
