# Supplementary material for: Identification of 7α,24-dihydroxy-3-oxocholest-4-en-26-oic and 7α,25-dihydroxy-3-oxocholest-4-en-26-oic acids in human cerebrospinal fluid and plasma
Source: Biochimie. 2018 Oct;153:86–98. doi: 10.1016/j.biochi.2018.06.020 (PMC6171785; doi:10.1016/j.biochi.2018.06.020)
Supplement: Multimedia component 1 [file mmc2.pdf]

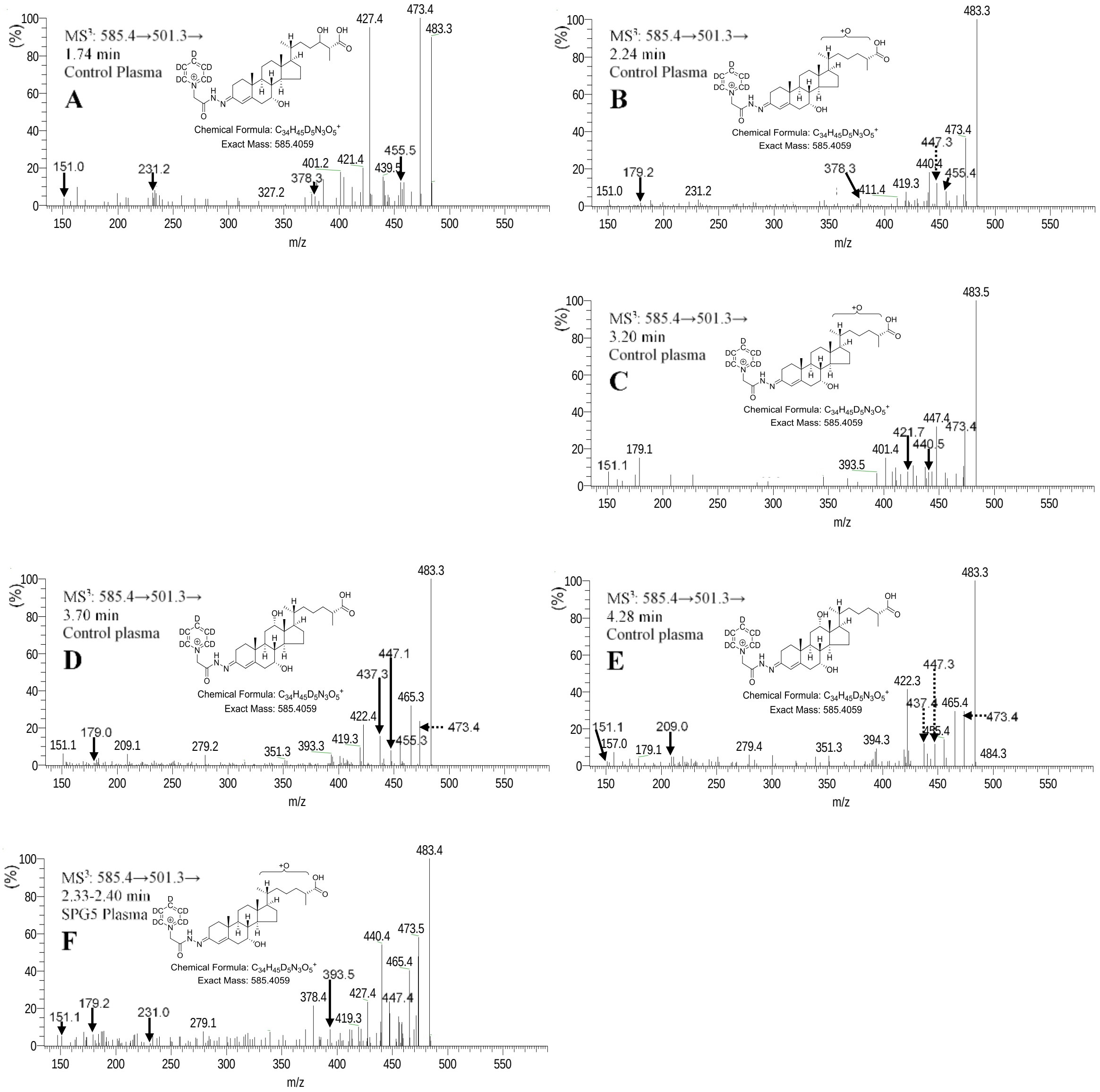


**Figure S8.** $MS^3$ spectra acquired in analyses of control (A-E) and SPG5 (F) plasma samples shown in chromatograms displayed in Figure 8. The spectra confirm the presence of dihydroxy-3-oxocholest-4-en-26-oic acids in plasma. (A) $CA^4$-7α,24-diol-3-one, (B & C) *syn* and *anti* conformers of $CA^4$-7α,x-diol-3-one, and (D & E) *syn* and *anti* conformers of $CA^4$-7α,12α-diol-3-one from control plasma, (F) $CA^4$-7α,x-diol-3-one in SPG5 plasma. Data was acquired in the LIT analyser of the Orbitrap Elite hybrid instrument with an accuracy of m/z ± 0.1 for most fragment-ions.

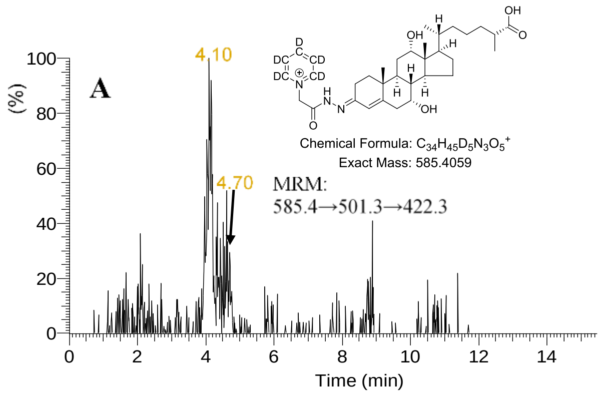


**Figure S9.** LC-MS$^3$ MRM chromatogram highlighting the CA$^4$-7α,12α-diol-3-one structure in plasma from CTX patient. The corresponding LC-HRMS chromatogram is shown in Figure 8C. Data was acquired in the LIT analyser of the Orbitrap Elite hybrid instrument with an *m/z* window of ± 0.4. Retention times were correlated to a QC sample analysed within the same batch of samples.

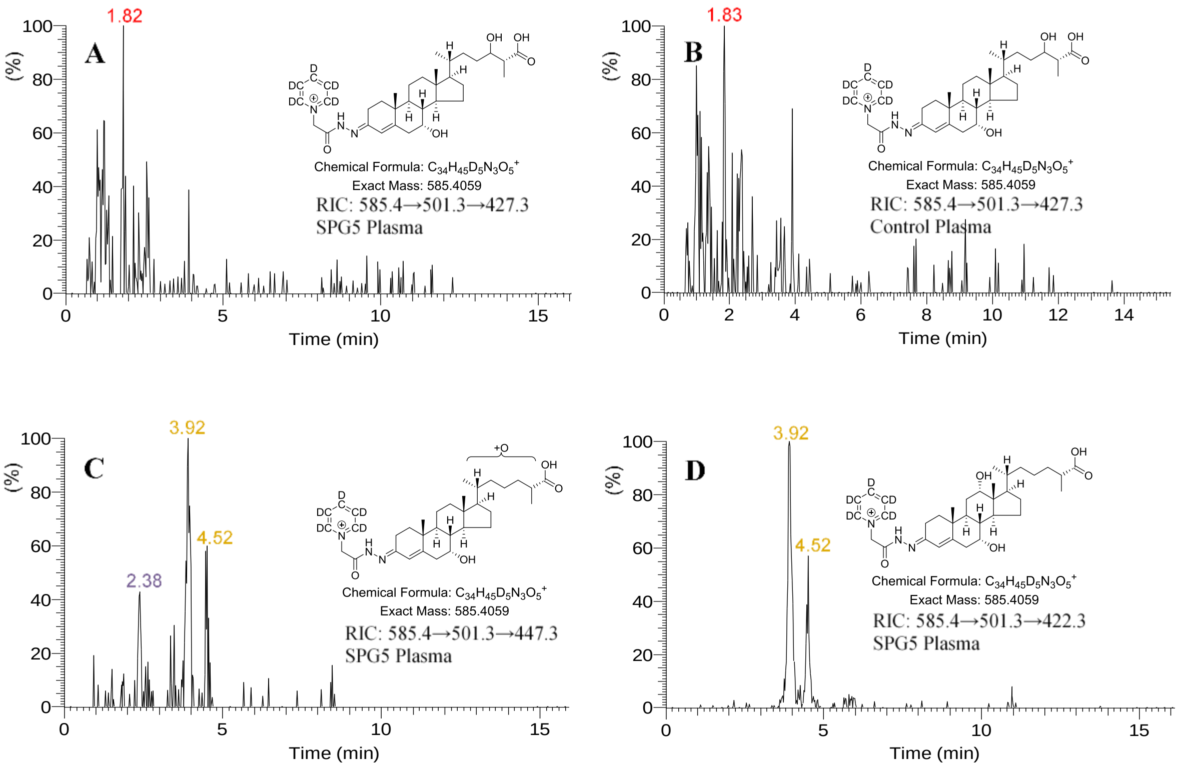


**Figure S10.** LC-MS[3] MRM chromatograms highlighting (A & B) CA[4]-7α,24-diol-3-one, (C) CA[4]-7α,x-diol-3-one, and (D) CA[4]-7α,12α-diol-3-one. (A, C & D) are from the analysis of SPG5 plasma and (B) is from the analysis of a QC sample. The corresponding LC-HRMS chromatogram for SPG5 plasma is shown in Figure 8D. Data was acquired in the LIT analyser of the Orbitrap Elite hybrid instrument with an *m/z* window of ± 0.4. Retention times were correlated to a QC sample analysed within the same batch of samples.
