# Supplementary material for: Identification of 7α,24-dihydroxy-3-oxocholest-4-en-26-oic and 7α,25-dihydroxy-3-oxocholest-4-en-26-oic acids in human cerebrospinal fluid and plasma
Source: Biochimie. 2018 Oct;153:86–98. doi: 10.1016/j.biochi.2018.06.020 (PMC6171785; doi:10.1016/j.biochi.2018.06.020)
Supplement: Multimedia component 1 [file mmc3.docx]

Supplemental Table S1. Mutation data, where available, for CTX and SPG5 patients providing CSF samples.

| Sample | Mutation |
| --- | --- |
| CTX:C | Heterozygous CYP27A1 c.666_678delCGAGAAACGCATT  c.1435C>T/p.R479C |
| CTX:D | Homozygous CYP27A1  c.1435C>T/p.R479C |
| CTX:E | Heterozygous CYP27A1  c.475C>T/p.Q159X  c.1214G>A/p.R405Q |
| SPG5:A | Homozygous CYP7B1  c.1456C>T/p.R486C |
| SPG5B: | Heterozygous CYP7B1  c.250delC/p.L84FfsX6  c.266A>C/p.Y89S |
| SPG5:C | Homozygous CYP7B1  c.825T>A/p.Y275X |
